# Extremozyme-Based Biosensors for Environmental Pollution Monitoring: Recent Developments

**DOI:** 10.3390/bios14030143

**Published:** 2024-03-14

**Authors:** Cristina Purcarea, Robert Ruginescu, Roberta Maria Banciu, Alina Vasilescu

**Affiliations:** 1 Department of Microbiology, Institute of Biology Bucharest of the Romanian Academy, 296 Splaiul Independentei, 060031 Bucharest, Romania; cristina.purcarea@ibiol.ro (C.P.); robert.ruginescu@ibiol.ro (R.R.); 2 International Centre of Biodynamics, 1B Intrarea Portocalelor, 060101 Bucharest, Romania; rbanciu@biodyn.ro; 3 Department of Analytical and Physical Chemistry, University of Bucharest, 4-12 Regina Elisabeta Blvd., 030018 Bucharest, Romania

**Keywords:** enzyme, biosensor, extremophile, environmental monitoring, catalytic activity, enzymatic inhibition

## Abstract

Extremozymes combine high specificity and sensitivity with the ability to withstand extreme operational conditions. This work presents an overview of extremozymes that show potential for environmental monitoring devices and outlines the latest advances in biosensors utilizing these unique molecules. The characteristics of various extremozymes described so far are presented, underlining their stability and operational conditions that make them attractive for biosensing. The biosensor design is discussed based on the detection of photosynthesis-inhibiting herbicides as a case study. Several biosensors for the detection of pesticides, heavy metals, and phenols are presented in more detail to highlight interesting substrate specificity, applications or immobilization methods. Compared to mesophilic enzymes, the integration of extremozymes in biosensors faces additional challenges related to lower availability and high production costs. The use of extremozymes in biosensing does not parallel their success in industrial applications. In recent years, the “collection” of recognition elements was enriched by extremozymes with interesting selectivity and by thermostable chimeras. The perspectives for biosensor development are exciting, considering also the progress in genetic editing for the oriented immobilization of enzymes, efficient folding, and better electron transport. Stability, production costs and immobilization at sensing interfaces must be improved to encourage wider applications of extremozymes in biosensors.

## 1. Introduction

In the contemporary era, environmental pollution stands out as one of the most pressing challenges, demanding immediate global attention and the implementation of comprehensive solutions to safeguard the well-being of ecosystems and human health. Many harmful substances, including pesticides, phenols, heavy metals, and polluting gases, are released and accumulate in the air, soil, and water due to the accelerated development of industries, rapid urbanization, and the burgeoning global population. Given that such pollutants have lasting negative impacts on all living systems, many countries have imposed stringent limits on the release of specific chemicals into the environment [1,2]. Nevertheless, the enforcement of this legislation requires reliable methods for monitoring toxic compounds.

Typically, the monitoring of pollutants relies on conventional chromatographic and spectroscopic methods, such as high-performance liquid chromatography (HPLC), gas chromatography (GC), and inductively coupled plasma-mass spectrometry (ICP-MS) [3]. While these analytical techniques offer high accuracy and sensitivity, they come with drawbacks, including labor-intensive processes, time consumption, the use of toxic chemicals, and the need for qualified personnel. Along with the tremendous advances in the field of nanotechnology, various chemical sensors relying on carbon nanomaterials, metallic, metal oxide or magnetic nanoparticles, metallic organic frameworks, covalent organic frameworks, etc., have been proposed for the detection of pollutants. While being fast and portable, they generally lack the required selectivity for analyzing complex samples. In response to these limitations, numerous endeavors have focused on the development of advanced biosensing devices capable of quickly detecting toxic substances in situ. These devices are generally designed to be selective, sensitive, and cost-effective, addressing challenges posed by traditional monitoring methods [3]. In biosensors, nanomaterials are integrated as (i) carriers for the bioreceptors, (ii) modifiers which improve the optical or electrochemical properties of the sensor or (iii) enzyme mimics (nanozymes).

Different types of biosensors have been reported in the literature for environmental monitoring [1,2,3,4,5,6,7]. These biosensors typically integrate whole cells or biological molecules (such as enzymes, aptamers, and antibodies) with a suitable physico-chemical transducer (such as electrochemical or optical) to produce a digital signal, proportional to the pollutant concentration. For instance, enzymatic biosensors utilizing acetylcholinesterase, choline oxidase, laccase, alkaline phosphatase, and urease operate based on the inhibition of these enzymes by a series of heavy metals, organophosphorus pesticides, and other organic and inorganic pollutants [8]. Similarly, biosensors based on whole microbial cells have demonstrated applicability in environmental monitoring due to their sensitivity to a range of chemicals [6]. The pollutants’ effect on natural biochemical processes (photosynthesis and bacterial respiration) and biochemical reactions provides a direct measure of a sample’s toxicity. Inhibition-based biosensors are valuable as screening and alert systems when analyzing complex environmental samples. In contrast, biosensors based on aptamers and antibodies determine specifically, with a high affinity for a single analyte. However, they do not provide information on the sample’s toxicity.

Among the various types of biosensors, those relying on enzymes as recognition elements are the most widely used due to their high specificity and sensitivity [8]. Nevertheless, the stability of enzymes remains a critical factor in applications demanding prolonged biosensor use. Harsh physicochemical conditions, including extreme temperatures, pH, and salinity, can induce protein denaturation, diminishing catalytic activity and impairing the overall functionality of the sensor. Consequently, substituting conventional mesophilic enzymes with homologs extracted from extremophilic microorganisms (often referred to as extremozymes) is revolutionizing the development of biosensors, ensuring enhanced stability for environmental applications [9].

Extremozymes represent a class of enzymes derived from microorganisms that thrive in extreme environments, such as hot springs, deep-sea hydrothermal vents, icy habitats, acidic lakes, soda lakes, or high-salt environments [10,11]. These enzymes have evolved to function optimally in extreme conditions, displaying remarkable adaptability and functionality in response to environmental stressors. Consequently, they can operate within a broad range of temperatures, pH levels, salinity, or hydrostatic pressure, offering significant advantages for applications in diverse environmental conditions [11,12,13].

Enzymes that have thus far demonstrated significant potential in developing biosensors for environmental monitoring include mainly oxidoreductases (e.g., laccase, tyrosinase, and peroxidase [1,8,11]), hydrolases (e.g., alkaline phosphatase, urease, and organophosphate hydrolase [1,8]), and the photosystem II (PS II) complex [5,8]. These molecules can be obtained from extremophilic microorganisms through two main approaches. Firstly, a culture-dependent method involves cultivating and isolating extremophilic microorganisms under the requisite physicochemical conditions. Subsequently, screening based on enzymatic activity is undertaken to identify the most promising extremozymes for further investigation. Secondly, a metagenomic approach relies on sequencing environmental DNA from extreme environments. This is followed by data mining to identify specific genes potentially encoding enzymes with valuable characteristics, which are then cloned and expressed heterologously in suitable hosts. Each method presents its own set of advantages and disadvantages, as discussed elsewhere [14].

In the past decades, several review papers have been published addressing the development of enzymatic biosensors for environmental monitoring [2,3,6], with some specifically focusing on the utilization of extremophilic microorganisms and their molecules in electrochemical systems and various industrial applications [1,11]. However, to our knowledge, no review has been published recently addressing the utility of extremozymes in biosensing devices for pollutant monitoring. In this context, the present work aims to review extremozymes with the most promising potential for environmental monitoring devices and present the latest advances in biosensors utilizing these unique molecules.

## 2. Extremozymes: Varieties and Significance

Using extremozymes in biosensors for pollutant monitoring offers several advantages that derive from the unique properties of these enzymes, including (1) enhanced stability in a wide range of physicochemical conditions, ensuring a longer functional lifespan for biosensors; (2) increased sensitivity in detecting pollutants, even in environments with fluctuating or extreme conditions; (3) broader applicability; and (4) cost-effectiveness due to the reduced need for replacements.

Among the various extremozymes found in nature, those showing activity and stability in wide ranges of temperature, salinity, and pH values are the most promising for developing robust biosensors for pollutant monitoring. A brief overview of these groups of enzymes is given below.

### 2.1. Cold-Active Enzymes

Microorganisms adapted to cold environments inhabit a wide range of low-temperature habitats, including Polar regions, high-altitude mountains, glaciers, ice caves, permafrost, and deep-sea environments [15]. The colonizers of these extreme environments comprise diverse species of bacteria, archaea, fungi, and other micro-eukaryotes. These microorganisms, which are characterized by an upper growth limit of 20 °C with optimal growth at 15 °C (psychrophiles) or are able to survive at low temperatures while also tolerating elevated temperatures (psychrotrophs) [16], present a series of molecular adaptations that allow them to function in cold conditions [17].

The enzymes produced by these organisms could efficiently function at lower temperatures due to their high flexibility, which reduces the reaction activation energy [18], thus representing novel performant biomolecules for various applications that demand cold conditions [17,19]. The structural features of the cold-active extremozymes encompass a reduced content of prolines, which are substituted by glycine, serine, or histidine residues that decrease the molecule rigidity [20], the presence of cysteine that improves the stability of the protein by disulfide bond formation, a low salt-bridge content [21], and a higher number of hydrophobic cores and surfaces [22] as compared with the mesophilic and thermophilic homologs [17].

In addition to a high activity at low temperatures, saving energy, several cold-active extremozymes are stable at temperatures significantly higher than that of their host environment, such as the recombinant aldehyde dehydrogenases (ALDHs) from *Flavobacterium* PL002 isolated from Antarctic seawater [23] and the marine psychrophilic *Cytofaga* sp. [24] that preserve their activity up to 60 °C. Also, microbial cold-adapted enzymes are easily isolated, heterologously expressed, and purified in large quantities, a critical advantage for producing these sensing biocomponents [25].

### 2.2. Thermostable Enzymes

Thermostable enzymes (thermozymes), derived from organisms that inhabit extreme environments such as hot springs, hydrothermal vents, or geothermal environments, can withstand and function efficiently at high temperatures [26,27]. The optimal temperature for these enzymes can range from 60 °C to 110 °C or even higher, depending on the source organism [27]. Based on their optimum growth temperature, these microorganisms belonging to bacterial, archaeal, or fungal species are classified as thermophiles (60–80 °C) and hyperthermophiles (80–122 °C) [27].

A series of specific structural features of thermostable enzymes prevent denaturation at high temperatures and maintain the enzyme’s activity [28]. These structural adaptations include a higher number of salt bridges and hydrogen bonds between side-chains, increased hydrophobic interactions, and a more rigid overall structure [29]. Most thermophilic proteins exhibit a higher content of Arg and Tyr residues and a reduced number of Cys and Ser residues and thermolabile residues (Asn, Gln) as compared to their mesophilic counterparts [30].

Besides their high thermal stability, thermozymes resist proteolysis and present a high chemical and pH stability [31,32]. Recent developments show that thermophiles are a major source of novel catalysts of great industrial interest. These extremozymes can replace mesophilic enzymes or chemicals in several industrial applications where high-temperature processes can reduce the risk of microbial contamination, improve the substrates’ solubility, and enhance the reaction rates. In addition, the heterologous overexpression of thermozymes in *Escherichia coli* allows the production of much larger quantities of enzymes, which are easy to purify by heat treatment [31].

### 2.3. Salt-Adapted Enzymes

Halophiles are a heterogeneous group of extremophilic organisms adapted to survival and even thriving in hypersaline habitats (i.e., >35 g/L of salts), which are inhospitable to most life forms on our planet. Representatives of this group are found in all three domains of life classification: *Archaea*, *Bacteria*, and *Eukarya* [33].

Maintaining the activity, stability, and solubility of proteins in hypersaline environments poses a true physiological challenge for halophilic microorganisms. The presence of a high salt content generally has negative effects on proteins, including: (1) the aggregation and collapse of tertiary structures due to intensified hydrophobic interactions, (2) interference with intra- or intermolecular electrostatic interactions between amino acids, and (3) the reduced availability of free water molecules due to the hydration of ions accumulated in cells. Therefore, to maintain functionality at very high salt concentrations, the proteome of halophiles must exhibit distinct structural characteristics from those of other physiological groups of microorganisms [34].

A common property of most proteins synthesized by halophilic microorganisms is their acidic nature. This characteristic results from a much higher content of amino acids with negative electric charge (glutamate and aspartate) than basic amino acids (lysine and arginine). Another structural property of most halophilic proteins is the much lower frequency of hydrophobic amino acids with long side chains (lysine, phenylalanine, isoleucine and leucine) than amino acids of small size (glycine, alanine, serine and threonine) [35,36,37]. Among salt-adapted proteins, enzymes demonstrate significant practical applicability. They can be employed to enhance the efficiency of biotechnological processes, including biosensing, in which hypersaline conditions result in the rapid inactivation of conventional enzymes [10,38].

### 2.4. Alkalistable Enzymes

Alkaliphilic microorganisms are divided into two primary groups: alkaliphiles and haloalkaliphiles. While alkaliphiles thrive at pH levels of 9 or higher, with an optimal growth pH of around 10, haloalkaliphiles necessitate both alkaline conditions (pH 9 or more) and high salinity (up to 5 M NaCl). They exhibit significant taxonomic and physiological diversity and have been found in a multitude of environments, ranging from neutral soils to extremely alkaline saline lakes [39].

Enzymes produced by alkaliphilic microorganisms showcase structural adaptations that allow them to function optimally in high-pH environments. These adaptations often involve an increased negative charge on the surface, altered hydrogen bonding networks, and enhanced stability of secondary structures [40]. These enzymes have found widespread applications in various industrial processes requiring alkaline resilience. Their significance extends to detergent formulations, wastewater treatment, and biofuel production, where their stability in alkaline environments proves invaluable for efficient and sustainable biotechnological processes [41,42].

### 2.5. Acidostable Enzymes

Acidophilic microorganisms are typically characterized by the ability to thrive in environments with a pH below 3, including acidic soils, acid mine drainage, acidic hot springs, and acidic water bodies. These microorganisms encompass a diverse range of bacteria, archaea, and eukaryotes that have evolved mechanisms to cope with and utilize acidic conditions for their growth and metabolism. They play a crucial role in biogeochemical processes, such as the cycling of minerals and metals [43].

Enzymes produced by acidophilic microorganisms have demonstrated activity and stability even at pH values as low as 1 [44]. Despite their remarkable performance under extreme acidity, the specific structural adaptations of these enzymes to acidic conditions remain unclear. A previous study [45] suggested that acid-stable enzymes may mitigate the challenges of low pH by reducing the density of positive and negative charges on the protein surface. This adaptation helps prevent electrostatic repulsion among charged groups, providing a potential insight into the resilience of these enzymes in acidic environments. Owing to their unique functional characteristics, acid-stable enzymes showed potential across various industrial and environmental applications, such as in the bioleaching of metals, the production of acidic beverages, and acidic wastewater bioremediation [46].

### 2.6. Extremozymes with Prospecting Applications in Environmental Monitoring

Although extremozymes possess unique characteristics that make them more advantageous than mesophilic counterparts in biotechnological processes, especially in applications where harsh physicochemical conditions would otherwise inhibit enzymatic reactions, they are generally not commercially available [11,47]. The large-scale purification and the reproducible production of extremozymes are challenging [14]. The limited number of commercially available extremozymes include a recombinant glutamate dehydrogenase from a thermophilic bacterium (available from Kerafast, Boston, MA, USA [48] and Athens Research and technology, Athens, GA, USA [47]) and a catalase from a psychrotolerant bacterium (produced by Swissaustral, Athens, GA, USA [49]). Additionally, thermostable laccases manufactured by Swissaustral LLC, which retain activity between 30 and 90 °C and at pH levels ranging from 5.5 to 7.5 [11], may find utility in environmental monitoring applications. Other extremozymes with potential utility in developing analytical methods for pollutant monitoring (including biosensors), such as laccases, tyrosinases, alkaline phosphatases, aldehyde dehydrogenases, and PS II, are presented in Table 1.

## 3. Characteristics of Extremozyme-Based Biosensors for Environmental Monitoring

### 3.1. Overview of Enzyme-Based Biosensors for Environmental Monitoring

Most enzyme-based biosensors developed so far have been intended for the biomedical field. At the same time, a smaller number have addressed food analysis and environmental monitoring [6,8,75,76,77], focusing on the detection of pesticides, biocides, dioxins, polychlorinated biphenyls, dyes, endocrine disruptors, heavy metals, toxins, drug residues, explosives, radiation, etc. [6,8,75,76,77].

The configuration, performances, and applications of enzyme-based biosensors were discussed in several recent reviews [6,8,78,79,80,81,82,83,84,85,86,87,88,89]. Both the direct catalytic activity and the inhibition of enzymatic activity were exploited in these applications [6,8,76,90]. Several factors are critical for achieving high analytical performance, including the enzyme characteristics, the mechanism of inhibition, the amount of enzyme, the incubation time, and the design of the biosensor (the immobilization matrix, any mediators, the electrode material, and nanomaterials) [6,8,78,79,90].

Recent examples of enzymatic biosensors applied for environmental monitoring are presented in Table 2, featuring devices based on glutathione transferase, acetylcholinesterase, butyrylcholinesterase, alkaline phosphatase, tyrosinase, glucose oxidase, peroxidase, xanthine oxidase, polyphenol oxidase, laccase, phosphotriesterase and organophosphorus hydrolase. The biosensor output consisted of an optical, electrochemical, or mass-sensitive signal.

### 3.2. Performances of Extremozyme-Based Biosensors and Bioassays

Extremozymes’ potential as biorecognition elements in biosensors has only begun to be tapped. Along with the capacity to withstand extreme environmental conditions, extremozymes have different substrate and inhibitor sensitivities, expanding the range of biosensor applications addressed by mesophilic enzymes.

The advantages of extremozymes immobilized in biosensors compared to devices based on mesophilic biocatalysts include the much higher sensitivity, increased resistance to organic solvents, and ability to operate at high temperatures for extended periods of time [110,111,112,113]. Among others, the larger detection sensitivity compared to the mesophilic counterpart was demonstrated for a thermophilic L-lactate dehydrogenase immobilized on a Au surface [112], for glucose-6-phosphate dehydrogenase cross-linked to an Os-based redox polymer on a graphite electrode [110], urate oxidase covalently immobilized on thioglycolic acid functionalized, a AuNP-modified glassy carbon electrode, etc. [113], to give just a few examples. Nonetheless, the major benefit remains the high stability of extremozymes (both storage and operational [111,113]), which is critical for advancing to commercial applications.

Unfortunately, as discussed in Section 2.6, a very small proportion of the commercially available enzymes are from extremophilic sources [114]. Additionally, the studies reporting new extremozymes were focused—in general—on preliminary characterization and did not include data on medium or long-term storage stability. Consequently, the low availability of extremozymes has limited the development of biosensors based on such biorecognition elements.

The extremozymes used in biosensors addressing various fields of application include native, recombinant, and chimeric proteins from different classes: oxidoreductases (which catalyze oxidation and reduction reactions), kinases (catalyzing the phosphorylation of different substrates) and hydrolases (catalyzing the hydrolysis of different chemical bonds) [10,115,116].

Concerning environmental monitoring, the applications developed until now have targeted the detection of pesticides, halogenated organic substances, phenolic contaminants, and heavy metals [116] (Table 3).

As emphasized by the data in Table 3, the biosensors for environmental monitoring based on extremozymes, developed in the past five years, specifically target the detection of photosynthesis-inhibiting herbicides, organophosphorus pesticides, and arsenic species. Most biosensors have relied on electrochemical transducers, detecting down to 10^−10^ M for the photosynthesis-inhibiting herbicide diuron. Nonetheless, very good detection limits have also been achieved by using fluorescence as the output signal, i.e., 10^−8^ M for the organophosphorus pesticide paraoxon. Critical aspects such as enzyme immobilization and selectivity of the obtained biosensors are commented on below, while design strategies and application of biosensors based on extremozymes are analyzed in Section 4 and Section 5, respectively. The biosensor design is discussed by taking as an example the detection of photosynthesis-inhibiting herbicides. This focus is justified by the fact that most extensive applications of extremophilic enzymes in biosensors concern photosynthetic preparations from thermophilic cyanobacteria. Photosynthetic systems have been extensively researched for both biosensing and photoelectrochemical cells for energy production. The organization of the sensing layer [146,147,148,149], the immobilization method and the use of artificial electron mediators were studied in detail for optimized electron transfer and photogenerated signals. Thus, a solid base of discussion exists for the discussion in Section 4. The applications of extremozyme-based biosensors are reviewed by discussing selected examples from Table 3. The aim is to underline the potential and some advantageous traits of extremozymes in the current analytical context.

### 3.3. Immobilization Methods Used with Extremozymes

The immobilization of enzymes on solid interfaces provides stabilization against degradation in harsh operational conditions, enables reuse and influences the enzyme’s catalytic properties and optimum operational conditions. It was achieved by various physical or chemical attachment strategies [150,151,152], in relation to the final purpose, e.g., inclusion in a bioreactor, reusable biosensor, biofuel cells, etc. Multipoint covalent interactions between enzymes and supports facilitated by glutaraldehyde, glyoxyl and epoxide groups lead to the adequate stabilization of enzymes for industrial applications [150]. It is a particularly effective approach for thermal stabilization, as demonstrated for esterase and catechol 2,3-dioxygenase from the thermophilic *Bacillus stearothermophilus.* The enzyme’s stability improved by 30,000-fold [153] and 700-fold [154], respectively, when immobilized on glyoxyl agarose beads as compared to the free enzymes. In biofuel cells, maximized power output is enabled by the oriented immobilization of enzymes on electrodes [155]. Immobilization conditions were often adapted to accommodate the attachment of multiple enzymes, for cascade reactions [156]. For example, four dehydrogenases from thermophilic microorganisms were immobilized in a Nafion membrane at the surface of a glassy carbon electrode, to build a bioanode exploiting the oxidation of L-proline [156]. The bioanode had 54% remaining activity after 15 days.

The immobilization of extremozymes onto a sensor surface is critical for the biosensor’s performance [157,158]. Suitable immobilization techniques should preserve enzyme activity, ensure stability, and facilitate easy regeneration of the biosensor. In biosensors developed in the last 5 years (Table 3), the biocatalysts were attached to membranes, electrodes or gold nanomaterials by physical adsorption, covalent immobilization by carbodiimide chemistry or by cross linking in either a matrix of BSA or in a redox polymer

The immobilization method influenced the affinity and sensitivity of the biosensor. For example, the apparent Michaelis–Menten constant of glucose-6-phosphate dehydrogenase from *Aquifex aeolicus* immobilized on graphite electrodes by cross linking in a redox polymer of osmium increased 16-fold, from 0.18 to 2.9 mM glucose-6-phosphate [110].

In addition to macro-sized supports, immobilization on nanomaterials provides further opportunities for obtaining stable biocatalysts with enhanced functional properties [159]. Compared to conventional immobilization techniques, nanomaterials offer notable advantages such as a large surface area, straightforward synthesis and high stability [160].

Covalent bonding of a mesophilic acetylcholinesterase on magnetic mesoporous silica nanoparticles improved both the stability in organic solvents and the sensitivity to pesticides [101]. The deposition of electrochemically reduced graphene oxide (rGO) and polyethyleneimine (PEI) on a carbon paper electrode facilitated the direct electron transfer of cellobiose dehydrogenase from *Myriococcum thermophilum*. The obtained bioanode had longer operational time, 48 h, as compared to only 24 h for an electrode lacking rGO [155]. There are numerous other examples of the nanomaterial-facilitated immobilization of enzymes. The range of immobilization approaches used with extremozymes is illustrated by a more detailed discussion in Section 4.2, in relation to the detection of photosynthesis inhibiting herbicides, taken as a case study.

### 3.4. Selectivity of Extremozyme-Based Biosensors

Most enzymes accept a wide variety of substrates. Enzyme-based biosensors display different degrees of selectivity, which is modulated by the sensor design and detection method (including the immobilization matrix, operational parameters, and type of enzyme) [161].

A biosensor based on, e.g., PSII, laccase, tyrosinase, acetylcholinesterase, aldehyde dehydrogenase, etc., applied to the analysis of environmental samples, which potentially contain mixtures of pollutants, including toxic pesticides and heavy metals, will provide a global response based on the contribution from all inhibitors to which the enzyme is sensitive. Such a “global toxicity” evaluation is very useful as an alert system. Cost-effective biosensors could be deployed to screen a large number of samples and alert when toxicity is detected. These assays enable us to limit the number of detailed, expensive analyses (e.g., by chromatography and mass spectrometry) to a few samples in which toxicity was detected. No matter how sophisticated and expensive a chemical analysis is, it cannot detect all chemical pollutants present in water samples [162].

The matrix or membrane used to immobilize the enzyme may act as a barrier for substrates or inhibitors from a certain class [99]. For example, the physical entrapment in gelatin of PSII particles from the thermophilic *Synechococcus elongatus* preserved the sensitivity of the immobilized photosynthetic preparations towards phenolic herbicides [118]. In contrast, by including PSII in a matrix of BSA via reticulation with glutaraldehyde, the inhibition of the photosynthetic activity of PSII caused by phenolic herbicides such as bromoxynil, ioxynil, and dinoseb was prevented for the most part. Owing to the different sensitivity of BSA–glutaraldehyde and gelatin gel-based biosensors, phenolic herbicides could be differentiated, in principle, from typical triazine (i.e., atrazine and simazine) and phenyl urea herbicides (i.e., diuron) [118]. Unfortunately, the above study did not include supporting data and a detailed investigation to substantiate the interesting effect of the immobilization.

The testing protocol is also a useful tool for controlling the selectivity of the enzyme biosensors. For example, a carboxyesterase from *Alicyclobacillus acidocaldarius* had a high affinity for paraoxon and methyl paraoxon, which caused fast and irreversible inhibition of the enzymatic activity. Instead, thio-organophosphate and carbamate pesticides did not significantly affect enzyme activity [163]. By converting the thio-organophosphate pesticides to their oxon form, these compounds irreversibly inhibited the enzyme with different kinetics. The different inhibition kinetics profiles can be recorded in minutes using an automated approach and a robotic workstation [126] and can serve as reference fingerprint traces. In perspective, these might be used by an approach integrating machine learning and multiple enzymes to identify the pesticides in a sample.

Temperature is another factor that might be exploited for controlling the selectivity of enzyme biosensors. Aoki et al. co-immobilized thermostable glucokinase and β-D-glucosidase on an ISFET sensor and demonstrated the detection of lactose at temperatures higher than 50 °C [139]. At this temperature, the lactose-hydrolyzing activity of the β-D-glucosidase was relatively high compared to the detection at 30 °C, where the activity was insignificant [139].

Additional opportunities to achieve higher selectivity when evaluating the toxicity of environmental waters [157] may be provided by (i) systems of multiple sensors, modified with enzymes having different substrate specificity and (ii) the inclusion of mutant bioreceptors, showing either resistance or, on the contrary, increased sensitivity to pollutants.

## 4. Design of Extremozyme-Based Biosensors: Case Study of Biosensors for Photosynthesis Inhibitors

### 4.1. Photosynthesis Inhibitors

Photosynthesis is the natural, highly efficient process that converts light into chemical energy and in which water and carbon dioxide are converted into oxygen and carbohydrates. The process takes place in the thylakoid membranes in cyanobacteria and in chloroplasts in plants. Two photosystems (PS), PSI and PSII, are located in the thylakoid membrane along with pigments, enzymes, and cofactors.

PSII is a dimer, each monomeric unit containing 20 protein subunits, 35 chlorophylls (Chl a), 11 carotenoids (b-Car), 2 pheophytins (Phe), 2 plastoquinones (primary, Q_A_ and secondary, Q_B_), 2 heme irons, 2 non-heme iron and a manganese–calcium (Mn_4_Ca) cluster (the oxygen-evolving complex, OEC) [148]. P680 is the primary electron donor of PSII, also called the reaction center (RC).

**Figure 1:**
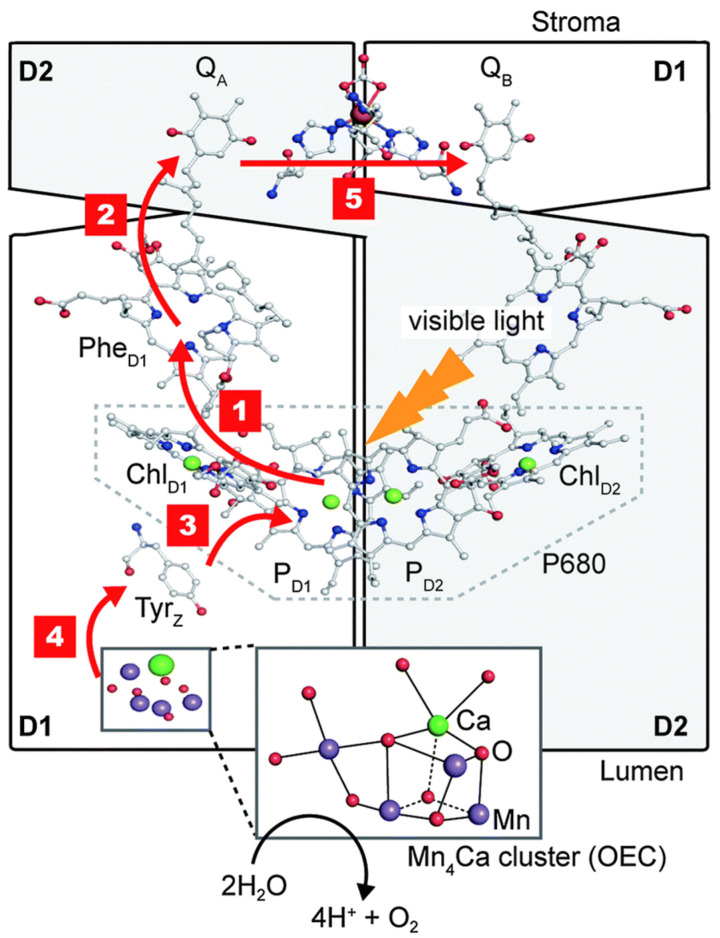
Schematic illustration of the reaction center RC complex in PSII showing the main components participating in the photosynthetic electron transfer as well the chronology of electron transfer steps in PSII (main steps are indicated by the numbers in a red frame). Reprinted from [148] with permission from the Royal Society of Chemistry.

In the first step, light excites PSII, which catalyzes the oxidation of water molecules at the OEC, producing O_2_ and protons [148]. Next, the electrons produced are transferred along the chain from PSII to PSI, passing to plastoquinone, cytochrome b6 f, and plastocyanin. The schematic illustration of the reaction center in PSII, (Figure 1) shows the 5-step process occurring in PSII: (1) upon excitation by light, charge separation occurs, resulting in the formation of P680^+^-PheD1^−^; (2) an electron is transferred from PheD1^−^ to primary plastoquinone Q_A_, forming Q_A_-; (3) an electron is transferred from tyrosine Z (Tyr Z) to P680^+^, forming TyrZ^+^;(4) the hole from TyrZ^+^ migrates to the Mn_4_Ca cluster; (5) an electron is transferred from Q_A_- to the terminal acceptor, the secondary plastoquinone Q_B_.

PSII is inhibited by herbicides, heavy metals, and several explosive compounds with chemical structures related to nitrophenols [164,165,166]. The herbicides that inhibit photosynthesis belong to two groups of compounds, binding to different sites located in the QB binding pocket in protein D1 in PSII of plants. Ureas, amides, triazines, triazinones, phenylcarbamates, pyridazinones, and uracils bind to Ser264, while benzothiadiazinones, nitriles, and phenyl-pyridazines bind to His215 [165].

Both optical and electrochemical methods were used to translate the inhibition of PSII into a quantifiable analytical signal. Chlorophyll a fluorescence is a very sensitive indicator of photosynthetic activity. Electrochemical methods measured either the production of oxygen or used electrochemical mediators to probe the electron transfer from Q_B_.

### 4.2. Configuration of the Sensing Layer

The attachment of photosynthetic materials to sensing interfaces to obtain highly performing and stable analytical devices was achieved by a wide range of procedures. Many studies aiming to identify the best biosensor design addressed the immobilization of PSII particles. The reason for this focus is that pure preparations with high photosynthetic activity are critical for both fundamental and more applied studies in energy production and sensing. PSII particles from thermophilic cyanobacteria, e.g., *Thermosynechococcus elongatus* and *Mastigocladus laminosus,* have adequate stability for such studies and provide a representative illustration of the extremozymes’ advantages. The immobilization methods applied to whole cells, thylakoids and plant chloroplasts and photosystems include the following:

- Physical procedures [167] by the
  (1) adsorption on filter paper, alumina, glass microfiber, or DEAE cellulose;
  (2) inclusion in polysaccharide (agar, agarose, carrageenan, alginate), protein (gelatin) or synthetic gels (polyacrylamide, polyurethane, poly(vinyl alcohol) and poly(vinylalcohol) functionalized with styrylpyridium groups, vinyl and photocrosslinkable resin);
  (3) adsorption on electrodes modified with conductive redox polymeric films;
  (4) layer-by-layer deposition of coatings with alternating positive and negative charge;
- Chemical immobilization by covalent [167,168] or non-covalent strategies [169].
  (1) the *non-oriented* covalent attachment of photosynthetic materials to sensing surfaces: amino groups from the lysine residues in the photosynthetic reaction centers (RC) were bound via bifunctional reagents such as glutaraldehyde to other amino groups in the other RC or in proteins such as bovine serum albumin, collagen, or gelatin, which protect the biological material against denaturation [152]. A special case is represented by the “wiring” of photosystems I and II to surfaces by cross-linking them to redox polymers. The polymer serves as both an immobilization matrix and as a facilitator of the electron transfer from the photosystem to the electrode surface.
  (2) the covalent, *oriented* immobilization of RC was achieved by chemisorption using the thiol group in the cysteine residue in the RC [168]. Alternatively, phosphonic acid linkers with carboxylic end groups were used to attract the PS by electrostatic interactions and the covalent bonds were formed via carbodiimide chemistry between the carboxylic groups of the linker and the amine groups in the RC.
  (3) *non-covalent*, *oriented* attachment of photosynthetic RC with engineered poly(His) tag to electrodes modified with a complex of nickel and nitrilotriacetic acid (Ni-NTA) via metal histidine affinity [169].

In the past years, most immobilization strategies used with photosynthetic preparations were intended for energy production and artificial solar cell applications, e.g., [170]. While the main goal was to achieve highly efficient electron transfer, i.e., large current densities, many immobilization methods used in these devices (Table 4) can be applied to biosensors.

Based on the data in Table 4, high current densities can be achieved by using hierarchically structured support interfaces, enabling high loadings of photosynthetic systems and redox polymers. For example, the amount of redox polymer immobilized on hierarchically structured inverse opal indium tin oxide (IO-ITO) electrodes was 50 times higher compared to flat electrodes [171]. Moreover, the use of the Os-based redox polymer leads to efficiently wiring the PSII to the electrodes, translated into large current densities [172], more than 10 times higher compared to electrodes modified with PSII alone [171]. Compared to PSII, thylakoids do enable larger current densities. Thin films of thylakoids wired to ITO nanoparticles via an Os-redox polymer at the surface of a graphite electrode enable higher current densities at lower overpotentials [173] compared to the simpler strategy of thylakoids adsorbed on carbon paper [174].

**Table 4:** Configurations used with photosynthetic preparations immobilized at electrodes for achieving high photogenerated current densities.

Biological Preparation	Immobilization Matrix	Applied Potential	Current Density (μA cm^−2^)	Reference
Thylakoid membranes from pea plants (*Pisum sativum* L.)	Adsorbed on carbon paper	1 V vs. Ag/AgCl	14	[174]
Thylakoid membranes from spinach	Thin film of thylakoids, osmium redox polymer, and ITO NP on a porous graphite electrode	0.4 V vs. Ag/AgCl	500	[173]
PSII and PSI from *Mastigocladus laminosus*	ITO	(a) PBV^2+^/PSI/PBV^2+/^PSI: −0.05 V vs. Ag/AgCl (b) PBV^2+^/PSII/PBV^2+^/PSII; 0 V vs. Ag/AgCl (c) PBV^2+^/PSI/PBQ/PSII, OCV	(a) 2.2 (b) 0.5 (c) 1.2	[175]
PSI extracted from baby spinach	PSI multilayer deposited	0.1 V vs. Ag/AgCl	7.9	[176]
	on electrode surface			
PSI from *Mastigocladus laminosus*	Bis aniline-crosslinked Pt NPs/PSI composite-modified surface	0.3 V vs. Ag/AgCl	4	[177]
PSII from *Thermosynechococcus elongatus*	PSII immobilized in Os^2+/3+^ complex containing hydrogel-modified electrode	0.3 V vs. Ag/AgCl	45	[172]
PSII from *Thermosynechococcus vulcanus*	His-tag/PSII-modified Au electrode	0.3 V vs. Ag/AgCl	2.4	[178]
PSII from *Thermosynechococcus elongatus*	Mesoporous ITO electrode	0.5 V vs. NHE	1.6 ± 0.3 2.2 ± 0.5 (45 °C) 12 ± 1 (with NQS) 22 ± 2 (with DCBQ)	[179]
PSII from *Thermosynechococcus elongatus*	IO-ITO-Os-based polymer–PSII IO-ITO-PSII	0.5 V vs. SHE	381 ± 31 33 ± 5 μA	[171]
PSII from *Thermosynechococcus elongatus*	MgAl-[FeCN)_6_]/PSII CoAl–[FeCN)_6_]/PSII	+0.5 V vs. NHE	0.07 ± 0.02 2.3 ± 0.2	[180]

Abbreviations: PEGDGE: Poly(ethylene glycol) diglycidyl ether. IO-ITO: hierarchically structured inverse opal indium tin oxide. PBV^2+^: poly N,N′-dibenzyl-4,4′-bipyridinium. PBQ: polylysine benzoquinone. OCV: open-circuit voltage. CoAl–[FeCN)_6_]/PSII, CoAl–[FeCN)_6_]/PSII: PSII drop-casted onto ferricyanide-intercalated cobalt–aluminum and magnesium–aluminum, respectively, layered double hydroxide. NQS: Potassium 1,4-naphthoquinone-2-sulfonate (DCBQ:2,6-dichloro-1,4-benzoquinone. ITO NP: indium tin oxide nanoparticles.

The photocurrent densities are enhanced by temperature and by the use of diffusional mediators [179]. In complex architectures, including multiple photosystems and polymeric electrochemical mediator layers, the ordering and the nature of the polymeric mediator film serving as adsorption layers for the photosystems determine the output of the final system [175]. There are various other sensing configurations besides the examples above, involving different electrode geometries and morphologies as well as different mediators that enable the efficient harvesting of energy from different photosynthetic preparations [170,181,182].

Nonetheless, for sensing purposes, there are additional performance indicators (besides maximized photogenerated current) that help in selecting a suitable immobilization procedure and configuration of the sensing layer. These indicators, discussed below, include (i) the stability of the biosensor, (ii) the selectivity and sensitivity to inhibitors; (iii) existence of alternative pathways for electron transfer, and (iv) the effect of the photosynthesis inhibitors on the stability of the sensing layer.

#### 4.2.1. Stability versus High Current Density and Short Response Time

The immobilization of PSII particles by cross-linking in a matrix of BSA using glutaraldehyde (BSA-GLU) provides good adhesion to electrode surfaces and leads to reproducible sensors. The immobilized PSII particles from the thermophilic *Synechococcus elongatus* had higher stability, i.e., a half-life of 8–24 h at room temperature, when compared to particles entrapped in different gels (agarose, alginate, or gelatin) [118]. The obtained biosensor displayed detection limits in the nanomolar range for diuron, atrazine, and simazine, whereas for phenolic herbicides ioxynil, bromoxynil, and dinoseb, the detection limits were in 10^−6^ M–10^−7^ M range. Cross-linking in BSA-GLU was a reference strategy in several studies examining alternative immobilization procedures [122,183].

**Figure 2:**
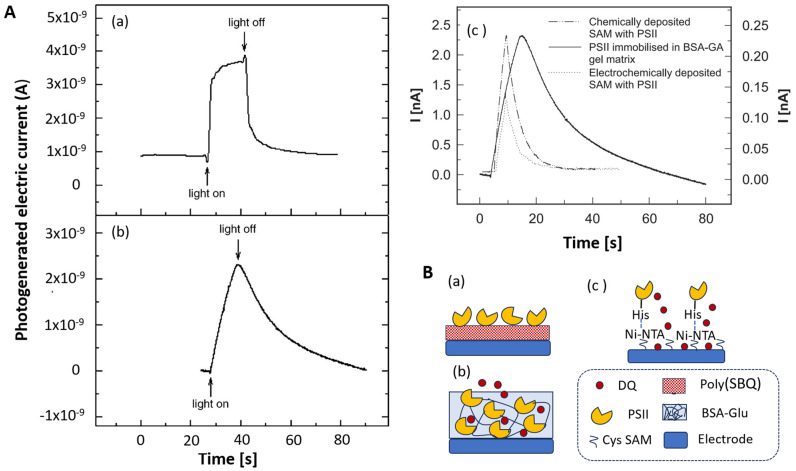
(**A**) (**a**): Photogenerated electric current obtained using PSII-polySBQ-AuWE screen-printed electrodes inserted in the flow chamber (40 mM MES buffer pH = 6.5, temperature = 5 °C, flow rate 0.25 mL min^−1^, WE potential *E* = +250 mV, illumination time = 10 s, illumination cycle = 180 s); (**b**) photogenerated electric current obtained using the PSII-BSA-GLU sensor configuration, as in [118]. Conditions are the same as in Panel (**a**). The artificial mediator duroquinone (DQ) is present in MES buffer solution (2 × 10^−5^ mol L^−1^). (**c**) current response of the PSII on Au WE. After illumination (5 s), the current increases due to the reoxidation of the artificial electron acceptor (duroquinone). Data obtained with three different immobilization methods on Au screen-printed WE [119]. Adapted from [122] for (**a**,**b**) and [119] for (**c**) with permission from Elsevier. (**B**) Schematic illustration of the biosensor configuration corresponding to the electrochemical signals shown in (**A**). Drawn based on descriptions in [122] (for (**a**)), [118] (for (**b**)) and [119] (for (**c**)).

Maly et al. immobilized PSII on a screen-printed gold electrode modified with a conductive, 30 nm thick layer of polymerized sulpho-p-benzoquinone [122]. The fast electron transfer from PSII to the polymer layer and very fast electrochemical reoxidation of the immobilized quinone in the polymer layer contributed to the short response time, as emphasized in Figure 2. The response curve obtained in the same experimental conditions with the “reference” PSII-BSA-GLU reveals the limitations to mediator diffusion in the immobilization matrix (from the shape of the electrochemical signal, increasing much more slowly upon illumination) (Figure 2).

Oriented immobilization of PSII from *Synechococcus elongatus* was accomplished by metal affinity binding between complexes of nitrilotriacetic acid with nickel ions attached at the electrode surface and histidine groups from poly(His)-tagged PSII [119]. The “reference” PSII-BSA-GLU was characterized by a response time of 15 min and IC50 of 9 × 10^−8^ M for atrazine. By comparison, a much faster response and improved IC50, i.e., 2 × 10^−8^ M, were obtained with His-tagged PSII, attached via Ni affinity to a Au SPE modified with a self-assembled layer of cysteine. A chemically self-assembled monolayer (SAM) of cysteine leads to a higher electrochemical signal than electrochemically deposited multi-layered cysteine, which leads to denser anchoring Ni-NTA points. Moreover, a mixed SAM layer of cysteine and octanethiol with increased hydrophobicity, “cysteine-OCT”, was claimed to lead to the best performance in terms of IC50 for atrazine, i.e., 5 × 10^−10^ M. However, the details regarding the preparation of the mixed SAM and the stability of biosensors in the different configurations were not provided. The differences in the sensitivity between the studied configurations of the sensing layer are due to the diffusional barriers for the electrochemical mediator according to each sensor architecture.

**Figure 3:**
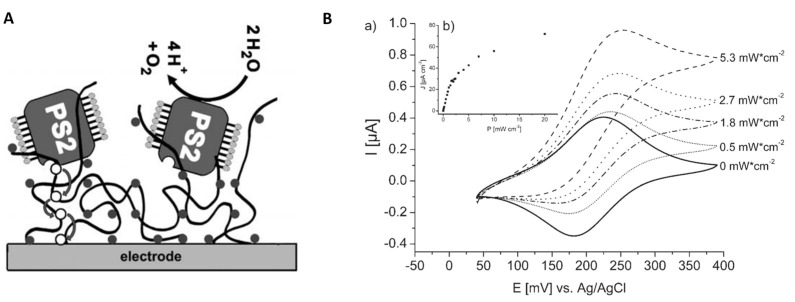
(**A**) PSII entrapped within a mediator-modified redox polymer. Arrows depict the electron transfer pathway by a hopping mechanism. (**B**) (**a**) Cyclic voltammogram of entrapped PSII complexes within a matrix of Os(bipy)_2_Cl-polymer and PEGDGE (full line). Dashed lines are representative CVs upon illumination with different light intensities (0.5–5.3 mW cm^2^) of the electrode at 675 nm. (**b**) Plot of obtained current from chronoamperometry vs. excitation intensity of a gold electrode modified with Os(bipy)_2_Cl-polymer, PEGDGE and PSII. Reproduced from [172] with permission from Wiley (Hoboken, NJ, USA).

Redox polymers were also investigated for the wiring of photosynthetic systems (PSII) to electrodes to achieve high photogenerated currents. For example, current densities up to 45 μA cm^−2^ were obtained with gold electrodes where PSII from *Synecococcus elongatus* were immobilized by reticulation in an Os(bpy)2Cl-modified poly(vinyl)imidazole redox polymer. Such current densities represent an increase by a factor of 10 compared to the case when PSII was simply adsorbed on an electrode modified with a redox polymeric film of poly(mercapto-p-benzoquinone) [122]. The polymer plays a double role: as an immobilization matrix for PSII and as an electron transfer facilitator (Figure 3A).

The redox polymer with reversible electrochemical behavior has a formal potential of +197 mV. It facilitates the fast electron transfer from PSII and eliminates the need for added electrochemical mediators to achieve high photogenerated currents from PSII. This sensing layer architecture allows for an appropriate density of PSII molecules and neighboring redox centers for efficient electron transfer within the polymer network down to the electrode surface, which is further translated into high current densities upon illumination (Figure 3B) [172]. Another advantage of entrapping PSII in this particular osmium-based redox polymer is the high stability under illumination, compared, e.g., to designs where PSII is immobilized at the SAM-covered electrode surface via Ni-NTA-His affinity, where the electron transfer to the electrode occurs via an exogenous mediator, e.g., 2, 6-dichloro-1,4-benzoquinone (DCBQ) [184].

Along the same line of research, the work of Kato et al. [185] was focused on finding a better method for the stable, covalent attachment of PSII from *Thermosynechococcus elongatus* in an oriented way at electrode surfaces. A nanostructured indium tin oxide (ITO) electrode served as the substrate material and was modified with a self-assembled monolayer (SAM) of phosphonic acid-linked compounds with amine or carboxylic end groups. SAMs with carboxylic groups (negatively charged) facilitated the oriented adsorption of PSII via electrostatic interactions. It has also enabled the covalent attachment of PSII by carbodiimide chemistry in a favorable conformation for fast electron transfer. While direct electron transfer was possible via short linkers of up to five carbon atoms, SAMs with longer linkers only allowed for mediated electron transfer from PSII, as demonstrated with the exogenous probe DCBQ.

**Figure 4:**
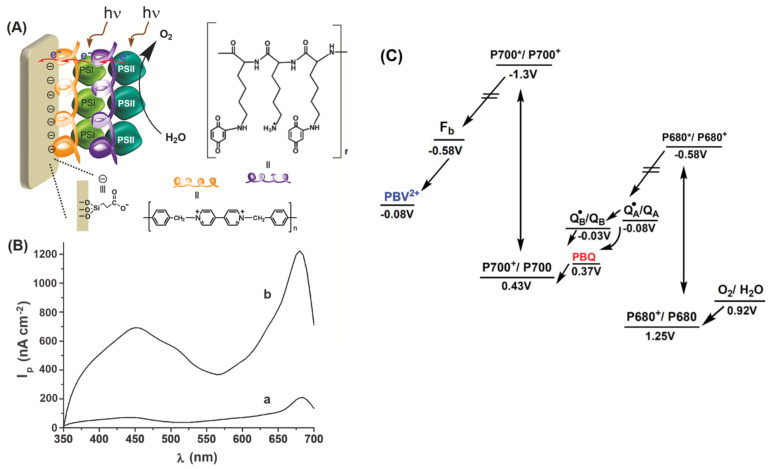
(**A**) Schematic assembly of the layered PBV^2+^/PSI/PBQ/PSII photoactive composite on an ITO electrode. (**B**) Photocurrent action spectra corresponding to (a) the PBV^2+^/PSI/PBV^2+^/PSII composite and (b) the PBV^2+^/PSI/PBQ/PSII composite. (**C**) Energy diagram for the cascaded electron transfer processes in the integrated PSI/PSII system containing PBQ (marked in red). The effective illumination area was 0.25 cm^2^. All measurements were performed in an Ar-deaerated phosphate buffer (0.1 M, pH = 7.2). Reprinted from [175] with permission from Wiley.

In another study, Yehezkeli et al. [175] assembled, on the surface of an ITO electrode, photobioelectrochemical systems in different configurations via layer-by-layer using PSI and PSII from *Mastigocladus laminosus*, and intermediate linking layers of redox polymers poly(N,N′-dibenzyl-4,4′-bipyridinium), i.e., poly-benzyl viologen, PBV^2+^, and poly(lysine benzoquinone), PBQ. The study revealed the importance of the order in which the photosystems were attached to the electrode for the direction of electron transfer within the coating and the possibility of tuning the biolayer configuration for obtaining an enhanced electron transfer. For example, anodic current intensities obtained with the PBV^2+^/PSI/PBQ/PSII system were six times higher compared to a simpler layer of PBV^2+^/PSII (Figure 4). The stability of these modified electrodes was around two days for PSI (activity decreasing by ca. 20% every 12 h) and less than one day for PSII (activity decreasing by ca. 25% every 10 h) when kept in the dark at 4 °C.

These studies reinforced the importance of sensor design for achieving fast response and high sensitivity. Many described biosensors cannot be efficiently regenerated to recover 100% of the initial value [122], i.e., one should consider these devices disposable. Recyclability issues should be considered when designing the biosensors, particularly when these include critical raw materials or noble metals.

Despite the fast response of the above-described sensors corresponding to more or less sophisticated configurations, most practical demonstrations with real environmental samples (soil and water) were performed with PSII systems from the thermophilic cyanobacterium *Synechococcus elongatus* f. *thermalis*, strain KOVROV 1972/8, chemically and stably immobilized in a matrix of BSA-GLU [118,120,121]. This reconfirms the sturdiness of BSA-GLU as an immobilization matrix for biomolecules. From another point of view, stability and application-specific data are simply missing for many sensing configurations, as the studies merely focused on achieving the highest possible photocurrent.

#### 4.2.2. Interfering Contributions to the Output Signal

To be accurate, a biosensor’s output signal should ideally reflect exclusively the contributions due to the measured analyte, without interferences. Nonetheless, when designing a biosensor for the detection of photosynthesis inhibitors, one has to consider the possibilities that (1) the sample will interact with the immobilization matrix in the sensing layer and that (2) additional electron transfer pathways exist, which circumvent the binding site of herbicides in the bioreceptors.

Some compounds may be repelled by the immobilization matrix while others enhance the stability of the enzymatic film and the intensity of the generated photoelectrochemical currents. Interestingly, it was found that among several inhibitors, dinoterb, a nitrophenolic inhibitor of PSII, provided higher stability and larger photocurrent intensity when incorporated in a redox polymer together with PSII from *Thermosynechococcus elongatus* [123]. Dinoterb, which has a non-polar tert-butyl group in the ortho-position, being moreover deprotonated at pH > 4.8, acts as a hydrophobic bulky counterion for the positively charged redox centers. This leads to the collapse of the polymeric film and a smaller distance between the redox centers, which ultimately translates into higher current intensities. Studies like [123], while rare, offer a fresh perspective for understanding the mechanism by which electrons are transferred from the immobilized photosynthetic systems to electrodes and how the composition of the immobilization matrix can be tuned for optimized detection performance [123].

The existence of alternative pathways for transferring electrons from photosynthetic systems to the electrode, bypassing Q_B_, was reported by several authors a long time ago and was investigated in relation to the sensor design and the electrochemical mediator [118,123,186,187,188,189].

**Figure 5:**
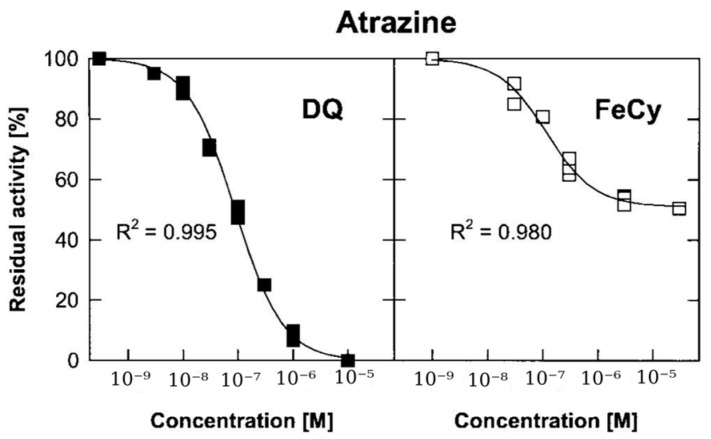
Calibration curves for atrazine in the presence of 0.2 mM DQ (left-hand panel) and of 1 mM FeCy (right-hand panel). The biosensor’s residual activity (in %) was calculated as the ratio of the signal obtained in the presence and absence of the herbicide. Adapted from [118], with permission from Wiley Periodicals, Inc. (2002).

In one of the first studies on this topic, Koblizek et al. developed a biosensor by immobilizing PSII from the thermophilic *Synechococcus elongatus* in BSA-GLU on a screen-printed graphite electrode [118]. The biosensor was tested with two diffusional mediators, duroquinone (DQ,) and ferricyanide (FeHEX (III) [118]. In the presence of high amounts of atrazine, i.e., 10^−5^ M, the total reduction in the response of the biosensor was observed when using DQ as a mediator (Figure 5). Instead, when using FeHEX(III) as a mediator, the measured signal plateaued at around 50% of the initial value at a high herbicide concentration. Corroborated with the observation that the affinity for atrazine (estimated by IC50) remained the same, the finding was explained by the existence of an alternative pathway for PSII reoxidation in the photosynthetic preparation. As it was insensitive to herbicides; it must have bypassed the Q_B_ binding site.

In a more recent work, the ability of an Os-based redox polymer to extract electrons from both the Q_A_ and Q_B_ sites of PSII was revealed by scanning photoelectrochemical microscopy (SPECM) [186]. The practical implication of these parallel pathways is lower biosensor sensitivity, as the blocking effect of herbicides on electron transfer is partially masked by the direct transfer from Q_A_ to the electrode [186]. Diuron and atrazine, as typical inhibitors that bind at the secondary quinone Q_B_ site, are used nowadays to emphasize the photocurrent due to the direct transfer from Q_A_ to the electrode. At the same time, studies with inactivated photosystems allow us to examine interfering, non-photosynthesis-related photocurrents [173] due, e.g., to the reduction in oxygen at chlorophyll pigments.

To summarize, the electrical wiring of photosystems to electrodes to transform light into electrical power and the assembly of natural photosystems to obtain photo-bioelectrochemical cells that mimic photosynthesis were intensively researched [181,190]. Besides subcellular preparations, whole cells of extremophilic microorganisms have also been studied to obtain stable preparations with high photosynthetic activity that can be available as lyophilized powder for practical applications in photo-bioelectrochemical cells [191]. Much of the knowledge obtained from such studies can be exploited for developing biosensors for photosynthesis inhibitors—a subject that attracted considerably less interest than energy conversion and production.

## 5. Applications of Extremozyme-Based Biosensors

The following section provides an overview of the applications of extremozyme-based biosensors for the detection of heavy metals, pesticides, and phenolic pollutants. Selected examples of biosensors from Table 3 are discussed in more detail, as they are representative for some traits of the extremozymes which may encourage wider use in biosensing.

### 5.1. Detection of Heavy Metals

Significant amounts of toxic metals found in waters, soils, sediments, and rocks are released by human activities in various fields such as mining, vehicle operation, industrial wastes, the dye industry, fertilizers, and batteries. The metals are found under various oxidation states and in various chemical combinations, with different degrees of toxicity [192]. To protect human health and the environment, MRLs for toxic metals in water and agro-food products have been established by regulatory agencies worldwide, and these are in the ppb–ppm range [193]. For example, the MRLs for inorganic mercury, arsenic, cadmium, lead, and copper in drinking water, according to Directive (Eu) 2020/2184 of the European Parliament and of the Council of 16 December 2020 on the quality of water intended for human consumption, are set in order at 1, 10, 5, 5 ppb and 2.0 ppm, respectively.

The quantitative analysis of heavy metals is typically performed by atomic absorption spectroscopy (AAS), inductively coupled plasma-mass spectrometry (ICP-MS), or energy-dispersive X-ray fluorescence spectrometry, as these methods have adequate sensitivity at ppb levels [192]. These laboratory-based techniques require skilled personnel and expensive equipment. Enzyme biosensors are attractive alternative analytical tools, being portable, fast and compatible with various detection techniques [80,83,194]. Numerous devices have been developed that exploit the inhibitory activity of metals on mesophilic tyrosinase, laccase, horseradish peroxidase, glucose oxidase, alkaline phosphatase, urease, nitrate reductase, etc. [82,97,103,195,196,197]. The catalytic activity of arsenite oxidase [198] and arsenate reductase [134] served, in addition, for the specific detection of arsenic species. Many biosensors are characterized by detection limits in the nanomolar range and a few of them even enable the detection of heavy metals in drinking water at ppb levels, compatible with the current MRLs [82].

Taking arsenic as a representative of toxic metals, it can be found in various forms, in both organic and inorganic compounds with different toxicities. While commonly found as (V), the As(III) is the form with higher toxicity. Enzyme-based biosensors based on mesophilic enzymes were developed based on the principle of enzymatic inhibition of, e.g., acetylcholinesterase and phosphatase [199,200], or based on the direct detection, e.g., the detection of arsenite using arsenite oxidase biosensors [198]. More recently, the detection of arsenic was addressed with an arsenate reductase extracted from *Thermus thermophilus* HB27 (TtArsC) [132,133,134] and with two thermostable protein chimeras [133].

**Figure 6:**
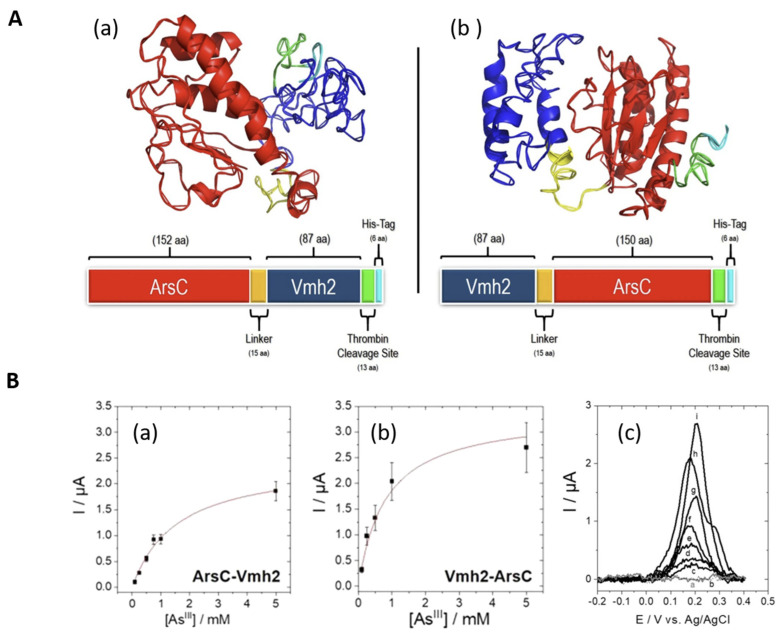
(**A**) 3D models of the chimeric proteins. (**a**) ArsC-Vmh2 and (**b**) Vmh2-ArsC. Different colors correspond to the following: Red: ArsC; Blue: Vmh2; Yellow: the linker; Green, the thrombin cleavage site; Cyan: the His-tag. (**B**) Plot of the SWV peak current versus As(III) concentrations for gold electrodes modified with (**a**) ArsC-Vmh2 and (**b**) Vmh2-ArsC. (**c**) SWV of the Vmh2-ArsC-modified gold electrodes after incubation (1 h, 60 °C) in solutions of 0.5 mM As(III) for the (a, gray) nonmodified gold electrode, and (b, dashed line) Vmh2-modified gold electrode; (c) 0.1, (d) 0.25, (e) 0.5, (f) 0.75, (g) 1, (h) 5 and (i) 10 mM As(III) for the Vmh2-ArsC-modified gold electrode. SWV parameter: 1 M HCl, pre-deposition at −0.4 V versus SCE for 5 min, scan rate 100 mVs^−1^, f = 50 Hz. Reproduced from [133].

The thermostable protein chimeras were obtained by the genetic fusion of Vmh2, a hydrophobin from *Pleurotus* ostreatus with self-assembling properties, and TtArsC, a thermophilic arsenate reductase from *Thermus thermophilus* HB27 [142]. The chimeras, Vmh2-ArsC and ArsC-Vmh2 (Figure 6A), displayed conserved arsenate reductase and phosphatase catalytic activity similar to the TtArsC and were immobilized on polystyrene beads and Au electrodes.

When attached to polystyrene beads and tested optically in microplates, both protein chimeras displayed a reusability of up to three times and storage stability of 15 days at 4 °C (i.e., expressed as a 50% decrease in phosphatase activity). The immobilized chimeras have almost five times higher specific activity compared to immobilized TtArsC. This indicates that the Vmh2 domain in the chimeras promotes efficient binding to solid surfaces with preservation of the enzymatic activity. Electrochemical biosensors for As(III) were obtained further by immobilizing the chimeras on gold electrodes. The immobilized chimeras are able to bind both As(V) and As(III). However, upon subsequent electrochemical reduction in an acidic medium (1 M HCl) on gold electrodes, only As(III) can be detected as it is readily reduced at As (0). The electrochemical reduction of As(V) to As (III) does not occur. It was found, moreover, that the affinity of the Vmh2-ArsC biosensor for As(III) was higher than that of the ArsC-Vmh2 biosensor, i.e., corresponding to association constants of 650 (±100) L·mol^−1^ and 1200 (±300) L·mol^−1^, respectively, at 60 °C and pH 7.5. The maximum current intensity for As(III) was also higher for Vmh2-ArsC (Figure 6B). These differences between the two chimeras were attributed to the more efficient immobilization and better accessibility of the substrate, As (III), to the catalytic site when Vmh2-ArsC was immobilized on the electrode. This proof-of-principle study emphasized the importance of protein chimera configuration and the potential of thermostable chimeras. Nonetheless, a further enhancement of the analytical performance is needed for practical applications that should address both the design of higher affinity chimeras and of more efficient transducers.

### 5.2. Detection of Pesticides

Modern agriculture relies heavily on the use of pesticides to achieve high productivity. The worldwide pesticide consumption in 2021 was 3.54 million metric tons, almost double compared to 1990 [201]. Meanwhile, the European Union is leading with respect to the number of banned highly hazardous pesticides, with 195 substances banned in 2022 [202]. Despite efforts to impose legislative measures to ban the very toxic compounds, to decrease the quantities used in agriculture [203], and to lower the maximum residue limits (MRL) admissible in agro-food products, pesticides will continue to be used extensively until effective alternative measures are identified. While the MRLs imposed by regulatory agencies worldwide, from the World Health Organization [204], the United States Environmental Protection Agency [193], and the European Commission [205], differ, these cover levels from below ppb to tens of ppm for pesticides in drinking water and agro-food products. For example, according to current Regulation (EC) 98/83/EC by the European Commission, MRLs in drinking water are set at 0.1 ppb and 0.5 ppb for individual and total pesticides, respectively. The analytical methods for detecting pesticide residues in various environmental matrices generally rely on chromatographic procedures coupled with mass spectrometry detectors. Often coupled with enrichment and purification procedures, these methods enable the detection of multiple analytes with adequate sensitivity and selectivity in line with the maximum allowed pesticide levels [206]. The detection of pesticides has advanced towards rapid and more cost-effective assays [207].

Enzyme-based biosensors have been proposed as alternative analytical tools to standardized chromatographic methods for the detection of pesticides along with antibody, aptamer, and molecularly imprinted polymer-based biosensors [83,207]. Most devices rely on the inhibitory effect of organophosphates, carbamates, dithiocarbamates, β-triketones, triazines, phenylureas, diazines, or phenolic pesticides on cholinesterases, photosynthetic system II, alkaline phosphatase, acid phosphatase, hydroxyphenylpyruvate dioxygenase, peroxidase, tyrosinase, laccase, urease or aldehyde dehydrogenase [7,76,82,93,109]. Several reviews have described the mechanism of inhibition, enzyme immobilization methods, detection strategies, and analytical performances of inhibition-based biosensors in detail, including [76]. A smaller number of devices for pesticide detection exploit the catalytic activity of organophosphate-degrading enzymes such as organophosphorus hydrolase [99], phosphotriesterase [93], or glutathione transferase [91]. This research field has advanced through the exploitation of nanomaterials, the use of mutant enzymes, miniaturized devices, and larger applications of chemometric sensors, showing a clear trend towards the development of simple and cheap, paper-based devices and detection via smartphones [6,8,76].

#### 5.2.1. Detection of Organophosphorus Pesticides

The continuous high interest in monitoring organophosphorus pesticides in the environment has been translated into a wide range of enzyme-based biosensors developed over time, based mainly on the inhibition of acetylcholinesterase, butyrylcholinesterase, laccase, tyrosinase or alkaline phosphatase and, in a much smaller measure on the degrading activity of organophosphate hydrolase and phosphotriesterase [8,101,208,209,210,211,212]. In the past years, nanozymes were included in the biosensor’s design to boost the detection sensitivity or have even replaced natural enzymes as the specific receptor in some devices, e.g., organophosphate hydrolase mimicking ceria nanoparticles or porous zeolitic imidazolate framework [209].

As an alternative biocatalyst, the thermostable esterase 2 (EST2) from *Alicyclobacillus acidocaldarius* is a carboxylesterase with high affinity towards paraoxon and methyl paraoxon. While being irreversibly inhibited by these compounds, is insensitive to other organophosphorus pesticides such as coumaphos, diazinon, dursban, fensulfothion, parathion and methylparathion [125,163]. The differences in selectivity between EST2 and the widely used acetylcholinesterase were associated with the tridimensional structure at the catalytic site of the two enzymes. Febbraio et al. suggested that steric hindrances may occur, depending on the chemical structure of the inhibitor [125].

**Figure 7:**
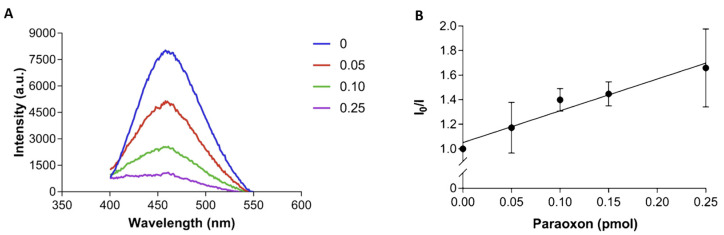
(**A**) Fluorescence emission spectra acquired with membranes containing 90 pmol of 1,5-IAEDANS-labeled EST2-S35C after excitation at 340 nm and (**B**) the corresponding ratios between the fluorescence intensity at the maximum of emission (462–468 nm) in the absence (I_0_) and presence (I) of increasing amounts of paraoxon (pmol). Reproduced from [129].

The enzyme displays remarkable stability, preserving its catalytic activity in the presence of detergents and at different temperatures. In a simple colorimetric biosensor, the recombinant enzyme was immobilized on a nitrocellulose membrane [125]. In another study, the recombinant enzyme, labeled with a fluorophore, 1,5-IAEDANS, was firmly attached by physical adsorption on a hydrophobic, polyvinylidene difluoride (PVDF) membrane [129]. The biosensor enabled the sensitive detection of paraoxon by fluorescence (Figure 7) down to 9 × 10^−8^ M in 1 min [129].

This example illustrates the potential of extremophilic enzymes, showing interesting selectivity (useful for sensor arrays or e-tongue devices), good sensitivity to pollutants, high stability and the conservation of catalytic function upon immobilization. The detection of organophosphorus pesticides is one of the most researched topics in biosensing and one of the most illustrative of the opportunities brought by the integration of advanced nanomaterials. Many highly performing devices were developed based on nanozymes [209], molecularly imprinted polymers [213], antibodies or aptamers, in addition to enzymes [214]. The current landscape of developed sensors and biosensors includes, e.g., (i) an electrochemical sensor with acethylcholinesterase, gold nanaoparticles (AuNP) and MoS_2_ nanosheets, with an LOD of 3.5 × 10^−11^ M for paraoxon [215]; (ii) an MIP sensor for the detection of fenamiphos based on a nanocomposite of core–shell Co_3_O_4_@MOF-74, with an LOD of 3.0 × 10^−12^ M, 60 days of stability and reusability of 50 times [216]; (iii) an aptasensor for malathion based on a cationic polymer and AuNP, with an LOD of 6 × 10^−14^ M [217]; (iv) an electrochemical sensor based on manganese dioxide nanosheets as a nanozyme, which achieved an LOD of 6.6 × 10^−10^ M paraoxon [218], etc.

It can be anticipated that by adopting new immobilization methods, nanomaterials and sensing procedures, the sensitivity of the above discussed extremozyme-based biosensor can be further improved. Nonetheless, for practical purposes, the advantages of a new device must be weighed against costs and stability features.

#### 5.2.2. Detection of Dithiocarbamate Fungicides

Dithiocarbamates (DTC) are widely used in agriculture to control fungal diseases in crops; e.g., mancozeb sales alone are forecasted to reach $18 billion by 2025 [219]. As these compounds can contaminate agro-food samples and the environment, maximum permissible limits were established in agro-food products; e.g., for the European Union, these range from 0.1 to 25 ppm [75]. The standard methods for analyzing these fungicides include gas chromatography and reverse-phase high-performance liquid chromatography with optical, electrochemical, or mass spectrometry detection [75]. Alternatively, electrochemical and optical sensors and several spectroscopy methods, in particular based on Surface Enhanced Raman Scattering (SERS), have been proposed. Nonetheless, as these alternative methods face selectivity issues, their coupling with specific bioreceptors is advantageous [75].

Several biosensors for the detection of DTC have been developed based on the inhibition of laccase, tyrosinase or aldehyde dehydrogenase [124,220,221,222,223,224], achieving detection limits in the ppb range [75]. Taking the example of aldehyde dehydrogenase, the NAD^+^-dependent enzyme catalyzes the oxidation of aldehydes to the corresponding carboxylic acids with the simultaneous reduction of the enzymatic cofactor NAD^+^ to NADH. The enzymatic activity and consequently, its inhibition by fungicides, is evaluated based on the quantity of formed NADH. Spectrometric, fluorimetric, or electrochemical methods can easily detect NADH. Recycling the cofactor by either the chemical or the enzymatic conversion of NADH boosts the sensitivity of fungicide detection as more aldehyde gets transformed. One approach is to couple two enzymatic reactions, while the simpler, cheaper alternative with electrochemical biosensors is to combine aldehyde dehydrogenase with electrochemical mediators for NADH ([124,222,223,224]). The enzymatic recycling of NAD^+^ can be achieved by coupling the NAD^+^-dependent enzyme with a diaphorase, NADH oxidase or a NADH-dependent dehydrogenase. A thermophilic NADH oxidase was successfully co-immobilized with aldehyde dehydrogenase by entrapment in a photocrosslinkable poly(vinylalcohol) bearing styrylpyridinium groups, at the surface of screen-printed electrodes. The obtained biosensors detected ppb levels of DTC [124].

Recently characterized enzymes from extremophiles may offer new opportunities for the detection of DTC. Preliminary data indicate adequate stability and preserved catalytic activity upon immobilization for aldehyde dehydrogenases from *Thermus thermophilus* [225] and from the Antarctic *Flavobacterium* PL002 [23], among others. The enzyme from *Thermus thermophilus* was immobilized by metal affinity to a Ni–agarose column in a flow reactor and was coupled with a reactor with L-lactate dehydrogenase, for the biocatalytic production of terephthalic acid [225]. The catalytic activity of the system decreased by only 7% after 7 days of storage, while the conversion rate of NAD^+^ was 63%. Another extremozyme, the F-ALDH aldehyde dehydrogenase from the Antarctic *Flavobacterium* PL002 [23], was sensitive to thiram and disulfiram [226] and retained significant catalytic activity when immobilized on a carbon nanofiber electrode [143].

New tyrosinases and laccases from extremophiles were also described (Table 1). While the stability and conservation of catalytic activity upon immobilization are important attributes, the sensitivity to DTC of all these recently described extremozymes remains to be established. There are also some potential limitations of biosensors based on enzymatic inhibition related to (i) selectivity and (ii) sample preparation, considering the low solubility of DTC. All the enzymes used for the detection of DTC are also inhibited by other pollutants, e.g., toxic metals, which may be present as well in the environment. In some cases, e.g., for detecting DTC on the surface of intact fruits and vegetables, SERS combined with artificial intelligence tools and “paste and peel” approaches provide the high-performance, fast and convenient detection of DTC [227], advantageous over any methods requiring tedious sample preparation.

#### 5.2.3. Detection of Photosynthesis Inhibiting Herbicides

**Figure 8:**
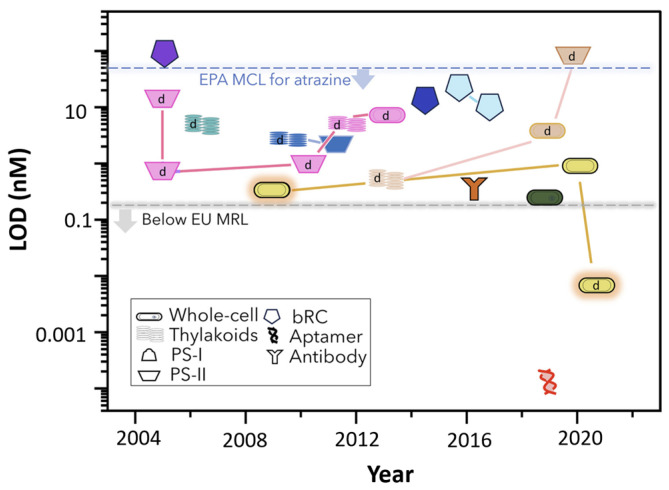
Developments in herbicide biosensors and their limits of detection, summarized in [229]. The different colors correspond to research groups contributing to this field, as detailed in [229]. The various shapes in the legend indicate the type of bioreceptor utilized in each study. Optical transduction methodologies are highlighted by a glowing red aura. The EU MRLs and EPA MCLs for atrazine are indicated in grey and blue dashed lines, respectively. The values reported are all for triazine-type herbicides, except for a few studies that utilized diuron, represented by a small ‘d’. EPA MCL = Environmental Protection Agency maximum contaminant level; EU = European Union’s maximum residue level. Adapted from [229] with permission from Elsevier (Amsterdam, The Netherlands).

Herbicides represent the majority of pesticides used in agriculture. About one third of commercially available herbicides have, as a mode of action, the inhibition of photosynthesis. The progress of biosensors for the detection of photosynthetic inhibitors (summarized in Figure 8) is described in detail in two illustrative reviews [228,229].

According to current data, similar sensitivity may be reached with either whole cells, thylakoids, bacterial reaction centers (bRC) or photosynthetic systems (PSI and PSII). Whole algal cells provide sensitivity and compatibility with different sensing formats [228,229]. Subcellular preparations including thylakoids, PSI, PSII, and bRC are sensitive, yet have limited stability, and their production is associated with high costs and low yields [228].

Many biosensors based on either photosynthesis inhibition or affinity (i.e., based on aptamers and antibodies) are appropriate for the detection of atrazine and other herbicides according to MRLs fixed by the EPA (Figure 8). A major recent advance in terms of the detection limit is the dual electrochemical–optical algal biosensor developed by Antonacci et al. [230], which, along with several aptamer-based biosensors for atrazine, meets the 0.1 ppb MRLs in drinking water imposed as per European regulations. The dual electrochemical and optical biosensor relies on whole cells of *Chlamydomonas reinhardtii* immobilized on paper-based, carbon-black modified screen-printed electrodes [230]. The detection of atrazine at trace levels down to 5 × 10^−12^ M is achieved by time-resolved fluorescence, while higher concentrations are detected by amperometry. By comparison, one of the most sensitive aptasensors developed so far has an LOD of 1.2 × 10^−12^ M [231].

The vast majority of the photosynthesis-based biosensors for environmental monitoring have been focused on monitoring aquatic environments using algae as bioreceptors. This is probably due to their relatively easy preparation by established protocols and lack of commercially available extremophilic preparations. The effort to develop analytical devices with enhanced stability, while minimizing the costs and environmental impact, is illustrated by, e.g., (i) a mediatorless electrochemical biosensor based on microalgae that retained 93% of initial activity after 5 months of storage at room temperature and detected atrazine in spiked river water [232]; (ii) an optical, environmentally friendly sensor obtained by immobilizing *Chlamydomonas reinhardtii* on a paper substrate for the detection of nanoformulated atrazine (herbicide), with a stability of 3 weeks at room temperature [233]; or (iii) a bioassay based on *Chlorella mirabilis* and chlorophyll fluorescence, integrated in an automated marine buoy that was operated by telemetry and used to detect herbicides in coastal waters [234].

An interesting example of an extremozyme-based biosensor applied for monitoring a herbicide in soil was developed by Maly et al., 2005 [120]. The biosensor, made by immobilizing PSII in a BSA-glutaraldehyde matrix on the surface of screen-printed platinum electrodes, detected isoproturon down to 10^−7^ M. The kinetics of isoproturon’s degradation in soil was followed at three depth levels. The concentrations of isoproturon determined with the biosensor were well correlated with those measured by standard methods, i.e., a growth assay and HPLC. Isoproturon was still detectable in the soil at 10 to 30 cm depth 9 weeks after application, whereas, in the top 0–10 cm layer, where the degradation was slower, the herbicide could still be measured after 11 weeks. Thus, the biosensor can be used as a cheaper and much faster method than HPLC to monitor the remaining levels in the soil. As a limitation, the measurement with the biosensor took 30 min. A remarkable fact about this study is that it highlights the degradation of herbicides in the environment, an issue that is largely overlooked in the biosensors field. Most bioreceptors—whether enzymes, antibodies, etc.—are exclusively characterized with respect to the detection of the original pesticide, while the degradation products formed upon its application in the environment are ignored, although these can be toxic as well. With the above-mentioned biosensor, lower inhibition was observed for the degraded isoproturon compared to the freshly applied compound. The sensitivity of the biosensor towards individual degradation products was not quantitatively evaluated with pure compounds. Nonetheless, the biosensor remains very useful to evaluate the persistence of toxic isoproturon degradation products in soil. Unfortunately, there are no recent similar studies of extremozyme-based biosensors for practical applications.

To conclude the applications of extremozymes in biosensors for the detection of pesticides, thanks to their different substrate specificity, these biocatalysts expand the analytical toolbox and the scope of the environmental alert systems that exploit the pesticides’ toxic effects on natural biochemical processes. The high stability of extremozymes enables the utilization of subcellular PSII preparations for both sensing and energy production applications. Older research like [120] is worth revisiting, given the progress in the nanomaterials field, to improve the analytical performances.

In perspective, the preliminary characterizations of several enzymes and extremophilic microorganisms, including mutants [235], pesticide-resistant bacteria [236] or newly characterized phototrophs [191] could serve as starting point for developing new biosensors in the future.

### 5.3. Detection of Phenols

Phenolic compounds are considered “priority pollutants”, having high toxicity and being widespread in the environment. The maximum admissible limit for total phenols in drinking water is fixed at 0.5 ppb (μg L^−1^), as per the United States Environmental Protection Agency. The specific determination of phenols is achieved by standard chromatographic methods coupled with mass spectrometry.

Additionally, the direct electrochemical oxidation of phenols on electrodes modified with carbon-based nanomaterials such as graphene, graphdiyne, carbon nanotubes, carbon dots, carbon black, biochar, etc. [237], is a cost-effective solution for the sensitive detection of phenolic compounds in well-characterized samples or simple mixtures. For more complex or unknown samples, to achieve the required selectivity, the nanomaterial-coated electrodes were either coupled with separation methods or used as support materials for enzyme-based biosensors. The specific detection of phenols with biosensors was achieved using tyrosinase, laccase, polyphenol oxidase, peroxidase or phenol hydroxylase as the recognition element [78,84,104,105,106,108,131,238,239], combined with advanced materials. Carbon-based nanomaterials, metallic organic frameworks, covalent organic frameworks, MXenes, and transition metal dichalcogenides have been used to enhance the sensitivity and selectivity of phenolic compound detection owing to their high surface area and high loading capacity with enzymes, superior conductivity or optical properties [237]. Moreover, some materials act as mimics of catechol oxidase, superoxide dismutase, horseradish peroxidase, laccase and hydrolase [237]. The combination of enzymes and nanomaterials have enabled us to achieve detection limits in the 10^−9^ M range, e.g., an LOD of 5.8 × 10^−9^ M phenol reported for a biosensor based on tyrosinase and nitrogen-doped graphdiyne-modified glassy carbon electrode [240].

Laccase was the most used enzyme and its immobilization and integration with nanomaterials were studied in detail, as the enzyme was also used for bioremediation and biotechnologies, in addition to sensing. Taking catechol as example, the detection limits achieved with laccase-based biosensors range from, e.g., 1.5 × 10^−6^ M to 8.5 × 10^−8^ M [240] and the stability reached 100 days at 4 °C [241].

Compared to these devices, the electrochemical biosensor based on phenol hydroxylase from the thermophilic *Bacillus stearothermophilus*, described in a 1999 report [131], detected phenol in the range from 2.5 μM to 400 μM. This is similar to some of the recent laccase-based biosensors. The thermophilic enzyme has a broad substrate specificity and, in contrast to the corresponding mesophilic enzyme, also accepts NADH as cofactor (much cheaper than NADPH). It also responds to 4-F-phenol and, to a lesser extent to 4-Cl-phenol but not to other para-substituted phenols. When used 8 h daily at 40 °C and stored at room temperature overnight, the biosensor lost 20% sensitivity after 7 days. Unfortunately, no data were reported for longer stability testing and no recent applications were developed with this enzyme.

To conclude, the combination of mesophilic enzymes with nanomaterials lead to stable and sensitive biosensors for the detection of phenols. Extremozymes, including, e.g., a recently reported thermostable laccase [242] might bring additional advantages in terms of selectivity and operational stability. Efficient immobilization strategies proven with mesophilic enzymes such as, e.g., attachment of *Trametes versicolor* laccase on sulfur-doped titania nanoparticles [243] or the electrospray deposition of the same enzyme [108], might find applications also with new bioreceptors from extremophilic strains leading to surface immobilized biocatalysts with high activity and stability.

## 6. Challenges and Perspectives of Extremozyme-Based Biosensors

Developing extremozyme-based biosensors can be technically challenging with limitations due to both the intrinsic properties of the biocomponent and the sensor development technology [239].

One of the challenges associated with the development of extremozyme-based biosensors addresses enzyme stability and activity under specific conditions in biosensor applications. Although these catalysts present enhanced functional features compared to other enzymes, the conditions under which biosensors operate (temperature, salinity, pH) may not be optimal for maintaining their activity over an extended period [244].

The light-induced, controlled activation of thermophilic enzymes immobilized on plasmonic nanomaterials [142,245], along with additional triggers of biocatalytic activity that exploit the interaction of enzymes with metallic nanoparticles [246], are innovative ways to maximize the activity and stability of extremozyme-based biosensors.

Moreover, biocatalysts’ stability could be enhanced through immobilization [247]. Stable attachment, combined with high catalytic activity is enabled by the interplay between the properties of the support material (structure, size, surface charge, hydrophobicity, porosity, and functional groups) and the characteristics of the biocatalyst enabling controlled, oriented immobilization. Using nanomaterials as support [158,160] and recombinant tagged-enzymes [248,249] for controlled immobilization on biosensors is an effective, versatile strategy for optimized enzyme immobilization. There are several solutions to prevent the loss of enzymatic activity during immobilization, including, e.g., nanosized organic–inorganic hybrids, created by combining metal phosphates as the inorganic component with enzymes as the organic component [189].

Additional efficient enzyme stabilization strategies could constitute the incorporation of additives such as ligands, salts, polyols, sugars, dimethyl sulfoxide, glycerol, polyethylene glycols, and synthetic polymers [250].

Protein lyophilization also represents a valuable technique to enhance the stability of extremozymes and their long-term storage by reducing their water content, thereby mitigating the risk of denaturation and degradation of the enzyme [251]. Optimization of this process requires various stabilizers, a personalized design of the lyophilization cycles to minimize stress on the enzyme and an advanced structural and functional characterization of the enzyme, providing insights into structural changes during this process [252]. The addition of various cryoprotectants (e.g., sucrose, trehalose, BSA, dextran, etc.) is a key factor for enhancing the overall efficiency of the lyophilization process, with preservation of all or most of its initial activity [69,253].

Another limitation in developing extremozyme-based biosensors is the production of sufficient quantities of purified enzyme. This usually requires the optimization of expression systems, which raises various technical and cost-effectiveness challenges [254]. In terms of cost and accessibility, the production of extremozymes may be more expensive than that of conventional enzymes [255]. Thus, developing profitable methods for large-scale production is a challenge in making extremozyme-based biosensors practical and commercially viable.

Furthermore, the selectivity and sensitivity of extremozymes for specific substrates are critical for the accurate and reliable detection of target analytes from complex samples, and this requires the selection of specific extremozymes based on particularities of environmental conditions [160]. Alternatively, these enzymes may need to be engineered for specific applications or conditions.

To address these challenges, a deep screening of unexplored extremophilic reservoirs could lead to the identification of promising enzyme candidates with enhanced properties for biosensing. Protein engineering, combining rational and computational design with directed evolution, can be used as a powerful tool for attaining and modeling the stability, selectivity and catalytic efficiency required in biosensing [14,256,257].

Recently, directed evolution has become a prominent strategy for enhancing enzyme stability and activity and modifying substrate specificity by systematically induced mutations in the enzyme’s genetic code, followed by a selection process to identify variants with improved characteristics [258]. This method has proven highly effective in discovering enzyme variants with novel or enhanced properties, such as modified substrate specificity or unique reactivity. In the past decade, genetically encoded biosensors (GEBs) have gained popularity in enzyme-directed evolution [259]. These biosensors translate the concentration of a small-molecule ligand into a readily detectable output signal. The integration of GEBs allows for the application of high-throughput techniques to evaluate the enzyme activity within extensive libraries for the highly efficient identification of variants with desired functionalities.

The utilization of recombinant extremozymes in biosensing not only allows for easy production of the sensor biocomponent in large quantities but also offers several advantages for developing improved biosensors. Recombinant tags can contribute to the stability of the immobilized enzyme by providing additional structural support. Thus, the quaternary structure of the enzyme is preserved and the lifetime of the biosensor is extended [260]. Some recombinant tags allow for easy regeneration of the biosensor surface [249]. This is beneficial for reusing the biosensor multiple times, reducing costs and improving the overall efficiency of the detection system. Furthermore, this system allows the binding of multiple recombinant enzymes with different specificities to the same biosensor, to simultaneously detect multiple analytes in a single assay [98]. Recombinant tags can be also designed to minimize nonspecific binding to the biosensor surface and enhance the signal-to-noise ratio of the detection system [261].

To have a broad image of the progress in enzyme-based biosensors and the main issues, it is useful to look at two literature reviews that are 10 years apart. Taking as an example the detection of photosynthesis-inhibiting pollutants, a 2010 study presented several promising biosensor-based platforms that were closer to technological maturity and were applied for the monitoring of environmental waters [262]. None of them relied on extremophilic enzymes. Currently, the number of biosensors dedicated to environmental applications remains very limited compared to applications in the biomedical field. The most relevant outstanding questions related to algae-based biosensing [263] also apply to the rest of the enzyme-based biosensors for environmental monitoring.

Advanced immobilization procedures, new materials (printed substrates, paper, nanomaterials), microfluidic and lab-on-a-chip (LoC) devices [263] can each contribute to enhancing the stability, sensitivity and selectivity of enzyme-based biosensors. Additional opportunities stem from the use of genetic methods (genome editing, site-directed mutagenesis), biomimetic receptors designed using computational tools, etc. Thanks to the rational design of artificial redox mediators, the electron transfer efficiency may be improved or the need for added redox probes may be eliminated by synthetic biology or protein engineering approaches [181].

Considering all the above, an open question is how to combine different technological and materials advances to really push the development of enzyme-based biosensors towards commercial devices [262]. Another challenge to overcome is to use these advances for the sustainable development of the biosensors while meeting strict requirements of cost-effectiveness, robustness and scalability [263].

In perspective, the promising extremophiles already used in biosensors as whole cells can be further purified and modified to obtain more sensitive extremozymes and extremozyme-based biosensors. These include, e.g., halophilic bacteria for heavy metal detection in saline water [264], acidophilic iron-oxidizing bacterium strain Y10 for heavy metal detection in acidic water [265], the thermophilic cyanobacterium *Gloeocapsopsis* sp. UTEXB3054 [191] or the halotolerant cyanobacterium *Aphanothece halophytica* [266] for studying photosynthesis inhibitors, etc.

## 7. Conclusions

Extremozymes are promising recognition elements in biosensors as they combine high specificity and sensitivity with the ability to withstand extreme operational conditions. In recent years, various extremozymes and thermostable chimeras with interesting substrate specificity have been isolated and characterized.

Despite their attractive characteristics, the use of extremozymes in biosensing does not parallel their success in industrial applications. A few biosensor applications cover the detection of pesticides, heavy metals and phenols. The majority rely on the principle of enzymatic inhibition and very few recent studies have addressed environmental monitoring.

The low availability of extremozymes provides an explanation for the low number of studies. Nonetheless, in many cases the stability of mesophilic enzymes was adequate; therefore, there was no incentive to look for alternatives. The reported extremozymes were not tested for long-term stability, nor were compared with mesophilic counterparts in similar operational conditions. Consequently, the potential of extremozymes is not entirely clear.

The main areas of interest with regard to environmental monitoring, concentrating most extremozyme-based studies, are represented by the detection of photosynthesis-inhibiting herbicides, organophosphorus pesticides and arsenic species.

Taking the detection of herbicides with electrochemical biosensors based on the inhibition of photosynthesis as a case study, several immobilization strategies, materials, and mediators for photosynthetic systems have been discussed. Advanced sensing configurations lead to high photocurrents generated by PSII-modified electrodes, as a result of efficient bioreceptor immobilization and the electron transfer pathway. Recent advances have been mainly due to sustained efforts for developing PSII-based photo bioelectrochemical cells for energy production, as such devices were enabled thanks to the adequate stability of purified PSII from thermophiles. The coating layer architecture of such devices cannot be simply translated into a biosensor design due to the possibility of including alternative electron transfer pathways insensitive to the presence of pollutants. Moreover, the interactions between the immobilization matrix and the targeted pollutant must be studied, as the matrix may act as a selective diffusion barrier for some pollutants, while other compounds may in fact stabilize the sensing layer.

The thermostable arsenate reductase and protein chimeras for arsenic species detection and the thermostable carboxyesterase for organophosphorus pesticide detection have different selectivities compared to the mesophilic enzymes typically used in biosensors. This may be advantageous for developing specific analytical devices. Carboxyesterase preserves its catalytic activity after being labeled fluorophore, while protein chimeras have good activity when immobilized on different surfaces, thus opening new sensing opportunities.

In addition, the preliminary characterization of several other extremozymes, used in simple assays or in other biosensors, paves the way for future applications in environmental monitoring. Given the sensitivity of luminescence detection, interesting applications may be facilitated by the recently reported extremozyme luciferases with good thermostability [267]. Pesticides and heavy metals inhibit the activity of the coupled system formed by NAD(P)H:FMN-oxidoreductase and luciferase, which is specific to luminescent bacteria [268]. Thus, new biosensors may be developed for the detection of the integral (i.e., “global”) toxicity.

The public interest in water and environmental monitoring is high. Considering that pesticide sales in Europe, e.g., have been at a constant level in recent years [269], and in view also of the recent disagreement at the European level regarding the lowering of pesticide use in Europe [204], it is envisioned that emphasis will be put more on the efficient use of all available measures including pesticides. In such a scenario, the monitoring of soil and water and development of adequate, fast, affordable analytical tools such as biosensors could become more important.

Compared to mesophilic enzymes, there are additional challenges for including extremozymes as recognition elements in biosensors, such as lower availability and high production costs. At the same time, the applicative potential of extremozymes in environmental monitoring is significant, taking into account that many of these enzymes were isolated from the same type of extreme or toxic environments as those that need to be analyzed.

New sensing strategies and materials working efficiently with the mesophilic enzymes are developed in parallel with the isolation and preliminary characterization of new extremozymes. So far, the interest in extremozymes for biosensing has been mostly related to their stability and selectivity, while one of their unique, valuable traits, i.e., their ability to operate in extreme environments, was not particularly exploited. Convergence of the advances in materials science and enzyme immobilization with the discovery of novel extremozymes may lead to biosensors operating in extreme environments or with superior characteristics, not achievable with mesophilic enzymes.

Genetically modified extremozymes with characteristics enabling oriented immobilization, efficient folding or better electron transport will bring additional advantages for designing high-performing biosensors. High stability, lower production costs, sustainable production, and efficient immobilization at sensing interfaces remain the main goals to encourage wider applications of extremozymes.

## Figures and Tables

**Table 1:** Examples of extremozymes with promising potential for developing robust environmental monitoring biosensors.

Enzyme	Source	Extreme Features	Potential Uses of Biosensor	Reference
Laccase	*Pycnoporus* sp.	Active at 0–100 °C (optimally 70 °C), at pH 4–10; half-life > 85 h at 60 °C	Detection of carbamate pesticides, dyes, and phenolic compounds in cold environments, such as marine areas	[50]
	Marine metagenome	Active at 0–70 °C (optimally 60 °C), at pH 4–8 (optimally pH 7); resistance to organic solvents and >1 M NaCl; stable > 2 h at 70 °C		[51]
	*Thermus thermophilus*	Optimal activity at 92 °C, at pH 5; half-life > 14 h at 80 °C	Detection of carbamate pesticides, dyes, and phenolic compounds in thermal basins	[52]
	*Thermobacullum terrenum*	Active at 70–90 °C, at pH 4.5; half-life > 2 days at 70 °C		[53]
	*Caldalkalibacillus thermarum*	Optimal activity at 70 °C, at pH 8; active at >1 M NaCl; resistance to various organic solvents, surfactants, and halides; half-life 12 h at 90 °C		[54]
	*Haloferax volcanii*	Active at 21–60 °C (optimally 45 °C), at pH 6–8.4; active and stable at >1.4 M NaCl; resistance to various organic solvents; half-life 31 h at 50 °C	Detection of carbamate pesticides, dyes, and phenolic compounds in hypersaline lakes, marine areas or wastewaters	[55]
	*Myrothecium verrucaria*	Active and stable at pH 8–11.5 (optimally pH 9); optimal activity at 70 °C; stable > 1 h at 50 °C	Detection of carbamate pesticides, dyes and phenolic compounds in wastewater treatment plants (alkaline) and mining areas (acidic)	[56]
	*Hortaea acidophila*	Active and stable at pH 2–7; optimal activity on ABTS * at pH 2		[57]
	*Proteus hauseri*	Optimal activity at pH 2.2, at 50–65 °C (optimally at 55 °C)		[58]
Tyrosinase	*Candidatus Nitrosopumilus koreensis*	Active at 0–60 °C (optimally 20 °C), at pH 5–8 (optimally 6); retained 50% of maximal activity at 0 °C	Detection of pesticides, hormones, and phenolic compounds in cold environments, such as marine areas	[59]
	*Thermomicrobium roseum*	Optimal activity at 70 °C and pH 9.5; retained 70% activity at pH 8.5–10; stable at <70 °C	Detection of pesticides, hormones, and phenolic compounds in thermal basins and hot industrial effluents	[60]
	*Symbiobacterium thermophilum*	Active at 50–80 °C (optimally 80 °C) and pH 6–9 (optimally pH 7); stable at pH 6–11 and at <80 °C		[61]
	*Streptomyces cyaneofuscatus*	Active at 50–70 °C (optimally 55 °C) and pH 5–10 (optimally pH 6.5–7.5); stable at 40 °C and pH 5–10		[62]
Alkaline phosphatase	*Shewanella* sp.	Active and stable at 0–80 °C (optimally 40 °C), at pH 6–11 (optimally pH 9.8)	Detection of heavy metals, pesticides and inorganic salts in marine areas, thermal basins, saline wastewater, etc.	[63]
	Antarctic strain TAB5	Active at 0–25 °C (optimally 25 °C), at pH 8.5		[64]
	*Thermotoga neapolitana*	Active at 20–90 °C (optimally 70 °C), at pH 7.5–11 (optimally 9.9); the half-life was 4 h at 90 °C		[65]
	*Thermus thermophilus*	Active at 40–95 °C (optimally 75–80 °C), at pH 8–12.5 (optimally pH 12); retained >50% activity after 6 h at 80 °C		[66]
	*Halomonas* sp.	Active at <2 M NaCl, at 37–50 °C and pH 6–11 (optimally pH 10.5)		[67]
	*Haloarcula marismortui*	Active and stable up to 3 M NaCl and KCl, at pH 7.5–10 (optimally pH 8.5)		[68]
Aldehyde dehydrogenase	*Flavobacterium* sp.	Active and stable at 10–40 °C (optimally 35 °C), at pH 7.5; resistance to various organic solvents and salts	Detection of dithiocarbamate fungicides in marine areas, thermal basins, saline wastewater, etc.	[69]
	*Anoxybacillus geothermalis*	Active and stable at 30–80 °C (optimally 60 °C), at pH 6–9 (optimally pH 8); tolerance to various organic solvents		[70]
	*Halobacterium salinarum*	Active at 0–3 M NaCl (optimally 1 M), at pH 7.2, and at room temperature		[71]
	*Natronomonas pharaonis*	Optimal activity at 60 °C, at pH 8, and at 0.25 M NaCl		[72]
	*Geobacillus thermoleovorans*	Optimal activity at pH 10, at 50–55 °C		[73]
Photosystem II	*Synechococcus elongatus*	Stabile for >21 days at 20 °C	Detection of herbicides, heavy metals, and endocrine-disrupting chemicals in marine areas and other environments	[74]

* ABTS = 2,2′-Azino-bis(3-ethylbenzothiazoline-6-sulfonic acid).

**Table 2:** Examples of recent enzyme biosensors for environmental monitoring.

Enzyme(s)	Analyte	Method	Analytical Characteristics	Sample	Reference
Glutathione transferase mutant Phe117Ile entrapped in sol-gel	α-endosulfan	Spectrophotometry	LR: 6.25 × 10^−7^–3 × 10^−5^ M	Spiked mineral and drinking water	[91]
Butyrylcholinesterase, alkaline phosphatase, and tyrosinase; origami paper device; carbon black modified screen-printed electrodes on office paper	Paraoxon, 2,4-dichlorophenoxyacetic acid (2,4-D), atrazine	CA	LOD: 2 ppb (paraoxon) 50 ppb (2,4-D) 10 ppb (atrazine) LR: 2–20 ppb (paraoxon) 100–600 ppb (2,4-D) 10–100 ppb (atrazine)	Spiked river water	[92]
Tyrosinase conjugated with carbon nano onions and immobilized in a chitosan matrix	Glyphosate	AMP	LOD: 6.5 × 10^−9^ M LR: 1.5 × 10^−8^–1.0 × 10^−5^ M	Water and soil samples taken from irrigation of a rice field	[93]
Glucose oxidase entrapped in chitosan on filter paper combined with SPCE	Cr(VI)	AMP	LOD: 0.05 ppm LR: 0.05–1 ppm	Water	[94]
Xanthine oxidase/cross-linking with glutaraldehyde/GC electrode	Bisphenol	AMP	Ki app: 8.15 × 10^−9^ M LOD: 1 × 10^−9^ M LR: 1 × 10^−9^ M–4 × 10^−8^ M	Mineral and river water	[95]
Mutant phosphotriesterase YT-PTE covalently immobilized on rGO, then drop-casted on SPCE	Organophosphates	DPV	LOD: 1.1 × 10^−7^ M	Spiked lake water, drain water, and soil run-off water	[96]
HRP and glucose oxidase co-immobilized in poly(noradrenaline) on a Pt electrode	Cr (VI) Cr (III) Glucose H_2_O_2_	AMP	LOD: 2 × 10^−10^ M (Cr(VI)) LOD: 1 × 10^−8^ M (Cr(III)) 8 × 10^−8^ M (glucose) 1 × 10^−5^ M (H_2_O_2_)	Water samples	[97]
Horseradish peroxidase entrapped in chitosan on filter paper	Catechol Resorcinol	Image analysis	LOD: 0.45 mM (catechol) LOD: 0.09 mM (resorcinol)	Water samples	[98]
Hexahistidine-tagged organophosphorus hydrolase immobilized on Zr-MOF (UiO-66-NH_2_)	Methyl parathion	Fluorescence	LOD: 10 ppb (3.4 ×·10^−8^ M) LR: 10–10^6^ ppb	Spiked tomato and orange	[99]
Phosphotriesterase cross-linked with glutaraldehyde on graphene electrode with Pt NP	Paraoxon	AMP	LOD: 3 × 10^−9^ M LR:1 × 10^−7^ M–1 × 10^−6^ M	Tap water, river water, soil slurry	[100]
Acetylcholinesterase covalently immobilized on magnetic mesoporous silica nanoparticles	Carbofuran Methomyl Isoprocarb Carbaryl	FL	LOD: 1 × 10^−8^ M LOD: 22 × 10^−8^ M LOD: 26 × 10^−8^ M LOD: 43 × 10^−8^ M	Spiked Chinese cabbage and cucumber	[101]
Choline oxidase immobilized in poly(brilliant cresyl blue)—on MWCNT/GCE	Dichlorvos	AMP	LOD: 1.55 × 10^−9^ M LR: 2.5 × 10^−9^ M–60 × 10^−9^ M	Spiked orange juice	[102]
Alkaline phosphatase covalently immobilized on SAM of 16-mercaptoundecanoic acid	Pb, Ni, Cd, Zn, Co, and Al	Nanocantilever	LOD: 0.32 ± 0.06 ppb (Pb) 0.87 ± 0.03 ppb (Ni) 0.33 ± 0.01 ppb (Cd) 0.48 ± 0.01 ppb (Zn) 0.42 ± 0.02 ppb (Co) 0.39 ± 0.01 ppb (Al)	River water	[103]
Polyphenol oxidase on filter paper	Catechol, phenol, p-cresol, 4-methyl catechol	Colorimetry/Image analysis	LOD: 5 × 10^−7^ M	River water Urine	[104]
Polyphenol oxidase entrapment in polyaniline-polyacrylonitrile-graphene hybrid and cross-linking with glutaraldehyde/Pt electrode	p-Cresol	AMP	LOD: 2.65 × 10^−7^ M (p-cresol) K_M_^app^: 2.53 × 10^−6^ M	Spiked river and sea water	[105]
Laccase mixed with BMIMBF4-and chitosan/MWCNT electrode	Bisphenol A	AMP	LOD: 8.4 ± 0.3 × 10^−9^ M LR: 0.5–12 × 10^−6^ M	River water	[106]
Laccase immobilized on L-cysteine functionalized-silver-coated magnetic Fe_3_O_4_ nanoparticles/SPE	Catechol	AMP	LOD: 6 × 10^−8^ M LR: 1 × 10^−7^–1 × 10^−4^ M	Lake and tap water	[107]
Laccase immobilized by electrospray deposition on SPCE	Catechol	AMP	LOD: 1.7 × 10^−6^ M LR: 2 × 10^−6^–1 × 10^−4^ M	Lake water	[108]
Acid phosphatase cross-linked with GA on a GO-AgNP/SPCE	Glyphosate	AMP	LOD: 15 ppb LR1: 50–500 ppb LR2: 500 ppb–22 ppm	Spiked water and soil	[109]

BMIMBF4: 1-butyl-3-methylimidazolium tetrafluoroborate. rGO: reduced graphene oxide. SPCE: screen printed carbon electrode. Zr-MOF: zirconium-based metallic organic framework. PtNP: platinum nanoparticles. GA: glutaraldehyde. GO-AgNP/SPCE: graphene oxide–silver nanoparticle composite electrodeposited on screen-printed carbon electrode; SWV: square-wave voltammetry. AMP: amperometry. DPV: Differential Pulse Voltammetry. FL: fluorescence. CA: chronoamperometry.

**Table 3:** Examples of biosensors and assays based on extremozymes addressing environmental monitoring, along with several recent applications from other fields.

Compound	Extremozyme	Technique	Analytical Characteristics	Reference
Diuron Atrazine Simazine Ioxynil Bromoxynil Dinoseb	PSII from *Synechococcus elongatus* immobilized on the surface of a Clark oxygen electrode by using a dialysis membrane	AMP	Diuron: LOD: 5 × 10^−10^ M I_50_: 8 × 10^−8^ M Atrazine: LOD: 2 × 10^−9^ M I_50_: 3 × 10^−7^ M Simazine: 1 × 10^−8^ M I_50_: 8 × 10^−7^ M Ioxynil: LOD: 9 × 10^−9^ M I_50_: 4 × 10^−7^ M Bromoxynil: LOD: 2 × 10^−7^ M I_50_: 1 × 10^−6^ M Dinoseb: LOD: 6 × 10^−8^ M I_50_: 8 × 10^−7^ M	[117]
Diuron Simazine Atrazine	PSII from *Synechococcus elongatus* cross-linked in a BSA matrix (BSA-GLU) or entrapped in a gel of agarose, alginate or gelatin; screen-printed graphite electrode	AMP	Immobilized in BSA-GLU: LOD: 1 × 10^−9^ M (diuron) LOD: 4 × 10^−9^ M (simazine) LOD: 2 × 10^−9^ M (atrazine)	[118]
Atrazine	His-tagged PSII from thermophilic *Synechococcus elongatus* immobilized on Ni-NTA/cysteine SAM without or with OCT or by cross-linking in BSA-GLU; Au screen-printed electrode	AMP	I_50_: 2 × 10^−8^ M (CYS–NTA–PSII) I_50_: 5 × 10^−10^ M (CYS + OCT) I_50_: 9 × 10^−8^ M (BSA–GA–PSII)	[119]
Isoproturon	PSII from *Synechococcus elongatus* cross-linked in BSA-GLU/Pt electrode	AMP	LOD: 9.1 × 10^−8^ M ED_50_: 2 × 10^−6^ M	[120]
Atrazine Isoproturon Diuron	PSII from thermophilic *Synechococcus elongatus* f. thermalis, strain KOVROV 1972/8, cross-linked into BSA-GLU-glycerol/Pt screen-printed electrode	AMP	LOD: 1.6 × 10^−9^ M LOD: 9.9 × 10^−9^ M LOD: 1 × 10^−9^ M	[121]
Diuron	PSII from *Synechococcus bigranulatus* strain Kovrov 1972/8 adsorbed on poly(SBQ)-Au electrode	AMP	LOD: 7 × 10^−10^ M I_50_: 9 × 10^−9^ M	[122]
Dinoterb, bromoxynil, 2,4-dinitrophenol	PSII from *Thermosynechococcus elongatus* immobilized by cross-linking in an Os-based redox polymer	AMP	Enhanced stability of the biosensor with herbicide co-immobilized with PSII, no inhibition	[123]
Ethylenebis(Dithiocarbamate) Fungicides Zineb, nabam	Aldehyde dehydrogenase from yeast co-immobilized with NADH oxidase from *Thermus thermophilus* on a Pt-sputtered screen-printed electrode	AMP	LOD: 8 ppb nabam LR: 10–80 ppb (nabam and zineb)	[124]
Organophosphorus pesticides	Esterase-2 (EST2) from *Alicyclobacillus acidocaldarius* immobilized on nitrocellulose membrane	COL/Image analysis	Detection of paraoxon in spiked fruit juices Detection range: up to 9 × 10^−7^ M paraoxon	[125]
Organophosphorus pesticides	EST2 in solution	FL	LOD: 205.5 ± 6.98 × 10^−15^ M (10% inhibition for paraoxon)	[126]
Organophosphorus pesticides	IAEDANS-labeled EST2-S35C (mutant of Esterase-2 from *Alicyclobacillus acidocaldarius* with the serine 35 replaced by a cysteine residue) developed to bind an extrinsic fluorescent probe	FRET	LOD: 2 × 10^−9^ M paraoxon	[127]
Organophosphorus pesticides	Recombinant EST2 from *Alicyclobacillus acidocaldarius* immobilized on a polyvinylidene difluoride (PVDF) membrane	FRET	LOD: 9 × 10^−8^ M LR: up to 3 × 10^−5^ M	[128]
Organophosphorus pesticides	IAEDANS-labeled EST2-S35C immobilized by physical adsorption on a polyvinylidene difluoride membrane	FL	Paraoxon: LOD: 9 × 10^−8^ M LOQ:3.1 × 10^−7^ M	[129]
Halogenated organic compounds	L-HADST (dehalogenase) from *Sulfolobus tokodai* covalently linked to N-hydroxysuccinimidyl Sepharose	POT	K_Mimm_ = 4.9 × 10^−3^ M K_Mfree_ = 4.9 × 10^−3^ M	[130]
Phenol	Phenol hydroxylase (EC 1.14.13.7) *Bacillus stearothermophilus* immobilized by sol-gel at the surface of a Clark type oxygen electrode	AMP	LR = 2.5 × 10^−6^–4 × 10^−4^ M	[131]
As(III) and As(V)	Arsenate reductase from *Thermus thermophilus* bound to PEG-stabilized Au NPs	UV-VIS y	LOD: 10 ± 3 × 10^−12^ M for As (III) and LOD = 7.7 ± 0.3 × 10^−12^ M for As(V)	[132]
As(III)	Thermostable chimeras Vmh2-ArsC and ArsC-Vmh2 immobilized on a Au electrode	SWV	AsrC-Vmh2: K_AsIII_ = 650 (±100) L·mol^−1^ buffer. Vmh2-ArsC K_AsIII_ = 1200 (±300)L·mol^−1^ at 60 °C in 50 mM Tris–HCl pH 7.5	[133]
As (V)	Arsenate reductase from *Thermus thermophilus*	COL	LOD: 0.28 ± 0.02 × 10^−6^ M	[134]
NADH	NADH oxidase from *Thermus aquaticus* immobilized on a Immobilon AV membrane	AMP	Measurement of lactate dehydrogenase activity in serum LOD: 2 × 10^−7^ M LR: 5 × 10^−7^–2 × 10^−5^ M	[135]
NADH	Diaphorase from *Bacillus stearothermophilus* immobilized on a Immobilon AV membrane at the surface of a Pt electrode; covered by a polycarbonate 0.33 μm membrane	AMP	Bienzymatic sensors (diaphorase and NAD-dependent dehydrogenase) for alcohol, lactate and β-hidroxybutyrate LOD (NADH): 5 × 10^−8^ M LR(NADH): 1 × 10^−7^–1 × 10^−3^ M	[136]
Glutamate	Glutamate dehydrogenase from the hyperthermophilic sulfur-reducing archaeon *Pyrococcus woesei*, immobilized in carbon paste with a polyethyleneimine- Toluidine Blue O redox polymer and lactitol/DEAE dextran	AMP	K_M_^app^ = 2.11–2.26 × 10^−3^ M at pH 7 and 45 °C K_M_^app^ = 1.17–1.32 × 10^−2^ M at pH 8.9 and 35 °C	[137]
Glutamate	Glutamate dehydrogenase from thermophilic archaebacterial isolate AN1 immobilized in carbon paste wax with toluidine blue O–acrylamide redox polymer and [Ru(NH_3_)_6_]Cl_3_	AMP(FIA)	LOD: 3 × 10^−4^ M LR: up to 4 × 10^−2^ M	[138]
Asparagine	Asparaginase from *Archaeoglobus fulgidus* retained on an ammonia ISE by a dialysis membrane	POT	LOD (L-asparagine): 6 × 10^−5^ M K_M_ (L-asparagine) = 8 × 10^−5^ M at 37 °C and pH 9.2	[111]
Glucose-6-phosphate	Glucose-6-phosphate dehydrogenase from *Aquifex aeolicus* cross linked in a redox polymer of osmium (1,10-phenanthroline-5,6-dione)2-poly(4-vinylpyridine on graphite electrodes)	AMP	LOD: 2 × 10^−4^ M LR: 6 × 10^−4^–2 × 10^−2^ M K_Mimmob_ = 2.9 × 10^−3^ M K_Mfree_ = 0.18 × 10^−3^ M)	[110]
Glucose-6-phosphate	Glucose-6-phosphate dehydrogenase from *B. stearothermophilus* cross-linked on a Pt black electrode; covered by cross-linked BSA film	AMP	LR: 1–50 × 10^−3^ M	[139]
Salicilin, esculin, cellobiose, lactose	Glucokinase and hydrolase from *Bacillus stearothermophilus* and β-D-glucosidase from *Caldocellum saccharolyticum* immobilized on a ISFET by cross-linking in BSA film	ISFET	At pH 7.0 and 40 °C. DR: 0.3–30 × 10^−3^ M salicilin Output (10 × 10^−3^ M solutions): Salicilin: 7.6 mV Esculin: 5.4 mV Cellobiose: 1.6 mV Lactose: <0.1 mV	[139]
D-proline	D-proline dehydrogenase from the hyperthermophilic archaeon *Pyrobaculum islandicum*, immobilized in agar on a GCE	AMP	Detection of D-amino acids in urine LR: 5 × 10^−4^–5 × 10^−3^ M D-proline K_M_^app^ = 7.9 × 10^−3^ M	[140]
L-lactate	L-lactate dehydrogenase from *Clostridium thermocellum* immobilized on Au electrode in a polyglutaraldehyde-polypyrrole film	AMP	Detection in blood LOD: 1.55 × 10^−4^ M Dynamic range: 4 × 10^−2^–1.6 × 10^−1^ M	[112]
Glucose	Recombinant Flavin Adenine Dinucleotide-dependent glucose dehydrogenase from *Talaromyces emersonii* immobilized on a SWCNT/PPF/Au electrode and covered by PPF	AMP	Detection of glucose in diabetic patients LOD: 5 × 10^−5^ M LR: 5 × 10^−6^–2.6 × 10^−2^ M	[141]
Aβ1-40 fibrils	Recombinant acylpeptide hydrolase from *Sulfolobus tokodaii*; complex with a single chain variable fragment (scFv 12B4), and gold nanorods	COL	Monitoring amyloid aggregation Theranostics	[142]
Benzaldehyde	Recombinant aldehyde dehydrogenase “F-ALDH” from *Flavobacterium* PL002 immobilized by cross-linking with GA in a BSA matrix on a CNF SPE	AMP (FIA)	Detection of benzaldehyde in benzoic acid raw material LOD:1 × 10^−5^ M LR: 3 × 10^−5–^3 × 10^−4^ M Km^app^ = 3.86 × 10^−4^ M Km^free^ = 1.45 × 10^−4^ M	[143]
Acetaldehyde	Recombinant aldehyde dehydrogenase “ALDS2” from *Flavobacterium PL002; CNT SPE*	AMP	Detection of acetaldehyde in wines LR: 1.25 × 10^−4^–2.5 × 10^−3^ M	[69]
Uric acid	Engineered urate oxidase from *Bacilllus* sp. TB-90 covalently immobilized on GCE modified with AuNPs and thioglycolic acid	AMP	LOD: 9.16 × 10^−9^ M LR: 5 × 10^−8^–1 × 10^−3^ M	[113]
Cellobiose	Cellobiose dehydrogenase from *Phanerochaete chrysosporium* cross-linked in a Os-based redox polymer with PEDGDE, on a GCE	AMPy	LOD: 2.55 × 10^−6^ M LR: up to 1 × 10^−4^ M Used together with a glucose biosensor to monitor the hydrolysis of cellulose and milled corncob	[144]
H_2_O_2_	Extremophilic fungus *Caldariomyces fumango* covalently immobilized on 3D-CNT@MoS_2_/IL_EM_/GCE	EC	LOD: 0.097 × 10^−6^ M LR: 0.2–997 × 10^−6^ M Detection of H_2_O_2_ in human urine	[145]

1,5-IAEDANS: 5-((((2-Iodoacetyl)amino)ethyl)amino) naphthalene-1-sulfonic acid. ISFET: ion-sensitive field effect transistor (ISFET). CNF-SPE: carbon nanofiber screen-printed electrode. CNT: carbon nanotube. SWCNT: single-walled carbon nanotube. PPF: plasma-polymerized film. PEGDGE: poly(ethylene glycol) diglycidyl ether. 3D-CNT@MoS_2_/IL_EM_/GCE: glassy carbon electrode modified with a nanocomposite of carboxylated carbon nanotubes with self-assembled molybdenum disulfide nanoflowers (MoS_2_) and the ionic liquid 1-ethyl-3-methylimidazolium bromide. OCT: octadecanethiol. AuNP: gold nanoparticles. BSA-GLU: matrix of bovine serum albumin cross-linked with glutaraldehyde. Poly(SBQ): electropolymerized film of poly sulpho-p-benzoquinone.ISE: ion-selective electrode; Vmh2: a hydrophobin from *Pleurotus ostreatus;* TtArsC, a arsenate reductase from *Thermus thermophilus*. EC: electrochemistry. AMP: amperometry. FRET: Fluorescence resonance energy transfer. POT: potentiometry. COL: colorimetry. FL: fluorescence. SWV: square-wave voltammetry. FIA: flow injection analysis.

## Data Availability

No new data were created or analyzed in this study. Data sharing is not applicable to this article.
